# Is generative artificial intelligence catalyzing a paradigmatic transformation in sport science? – Evidence from a mixed-methods systematic review

**DOI:** 10.5114/biolsport.2026.162050

**Published:** 2026-08-24

**Authors:** Shunfang Liu, Yongyu Huang, Yupeng Shen

**Affiliations:** 1 School of Physical Education and Sports Science, South China Normal University, Guangzhou, China

**Keywords:** Generative artificial intelligence, Sport and exercise science, Systematic review, Bibliometric analysis, Topic modelling

## Abstract

This mixed-methods systematic review synthesises current evidence on the application of generative artificial intelligence (GAI) in sport and exercise science. Following PRISMA guidelines, 64 eligible studies were identified from Web of Science, PubMed, SPORTDiscus, Scopus and other sources, and methodological quality was appraised using JBI critical appraisal tools. Evidence was integrated using bibliometric mapping, Latent Dirichlet Allocation topic modelling, and narrative synthesis. Findings indicate that GAI is increasingly applied across physical education, personalised training, exercise rehabilitation, and health promotion. Two dominant research trajectories were identified: (i) methodological evaluation of GAI-generated content and (ii) application-oriented deployment in real-world practice. Overall, GAI demonstrates moderate to good performance in content generation, guideline adherence, and decision-support efficiency, particularly under structured, high-information prompting. However, most studies remain evaluative or exploratory, with limited high-quality randomised trials, short intervention durations, and insufficient real-world validation. Persistent limitations include inadequate individualisation, instability across outputs, safety concerns in high-risk populations, and emerging ethical challenges related to data governance, bias, and accountability. In conclusion, GAI represents a promising adjunct tool in sport and exercise science, but current evidence is insufficient to support widespread implementation or paradigm-level change. Future research should prioritise rigorous experimental designs, clinically and performance-relevant outcome measures, domain-specific system development, and robust ethical governance frameworks.

## INTRODUCTION

Generative artificial intelligence (hereafter “GAI”) has advanced to a stage at which it can produce novel, user-directed content through continuous learning and the refinement of underlying logical patterns. Such outputs span multiple modalities, including text, images, and video [1]. Large language models such as OpenAI’s ChatGPT and Google’s Gemini are capable of generating lengthy, coherent, and stylistically sophisticated text [2]; generative adversarial networks can synthesise photorealistic images from textual descriptions [3]; and models such as OpenAI’s Sora and Stable Diffusion have further extended these capabilities by transforming text prompts into video sequences [4]. Sport and exercise science, by contrast, is inherently interdisciplinary, drawing upon physiology, biomechanics, psychology, medicine, and nutrition science [5] to enhance human performance and promote health across diverse practical settings—including training and performance analysis, rehabilitation, injury prevention, and public health. These application scenarios often involve complex multimodal data (e.g., movement-recognition streams, video analysis, physiological signals) and require highly individualised training and rehabilitation programmes. Such features create natural entry points for AI-driven tools while simultaneously revealing the field’s methodological and practical gaps.

GAI has already demonstrated transformative potential across sectors such as education and healthcare. In education, GAI has evolved far beyond traditional search tools or conversational interfaces; it is increasingly reshaping instructional design, learning processes, and assessment practices, and redefining the evolving roles of teachers and students [6, 7]. In healthcare, ChatGPT and related models have shown promise in supporting clinical decision-making, elder care, medication management, weight control, nutrition guidance, health promotion, scientific research, and medical education [8], underscoring their broad potential within healthcare delivery systems [9]. Despite these advances, the role of GAI in sport and exercise science remains comparatively underexplored. The field’s emphasis on personalised training prescription, scenario-specific problem-solving, and sensor-based data streams positions it as a unique testbed for GAI innovations; however, most existing studies remain limited to isolated case analyses or demonstrations of single technical functions. For example, Washif et al. showed that ChatGPT could generate a structured 12-week resistance-training programme that partially aligned with authoritative sources such as NSCA guidelines [10]. Meanwhile, GPT-4o was used to generate eight training plans, and ratings from 11 sport-science experts indicated that while model outputs were highly variable, detailed and structured prompts (incorporating FITT principles and individual characteristics) substantially improved the quality, scientific accuracy, and stability of the generated programmes [11]. Parallel explorations have applied GAI to personalised rehabilitation planning for conditions such as knee osteoarthritis [12, 13] and to improving patient engagement in the rehabilitation process [14]. Accordingly, there is an urgent need to systematically evaluate and integrate the cumulative evidence generated across these diverse lines of research, shifting from isolated application assessments to a comprehensive evaluation of the field’s overall utility, limitations, and paradigm implications.

Existing review studies have begun to delineate the contours of this emerging field. For example, the narrative review by Ha et al. examined ChatGPT applications across multiple sport science subdisciplines, including exercise science, physical activity promotion, physical education, and sport management, providing a broad overview of potential use cases and associated ethical considerations [15]. Puce et al.’s systematic review further reported that, although GAI-generated exercise prescriptions generally adhered to established guidelines, notable limitations remained in individualisation and progression-oriented feedback [16]. Similarly, Liu et al. synthesised evidence on large language model–based weight management interventions implemented under hypobaric and hypoxic conditions, suggesting that AI-driven personalised lifestyle interventions may improve health management efficiency and reduce chronic disease risk [17]. While informative, these reviews predominantly focus on discrete application domains or rely on conventional outcome-oriented evaluation approaches—often emphasising quantitative indicators such as prescription quality. As a result, they provide limited insight into the broader, system-level transformations introduced by GAI across the sport science ecosystem, including its potential implications for research methodologies, coaching practices, and ethical governance. More integrative syntheses are therefore required, not only to determine whether specific applications are effective, but also to clarify how and why GAI may be reshaping the underlying questions, methods, and evaluative standards of the field.

To address this gap, the present study adopts a mixed-methods systematic review design informed by the mixed research paradigm proposed by Pluye et al. [18]. Quantitative approaches, including bibliometric analysis and latent Dirichlet allocation (hereafter “LDA”) topic modelling, are used to map research hotspots, knowledge structures, and developmental trajectories at the macro level. In parallel, qualitative synthesis provides in-depth interpretation of practical challenges, ethical tensions, and applied implications of GAI in sport science contexts. Importantly, findings from the quantitative analyses guide the focus and interpretive framework of the qualitative inquiry, enabling a structured and purposeful integration of the two strands. Guided by this approach, our review aims to investigate whether and how GAI is catalyzing a paradigmatic transformation in sport science. Specifically, it addresses three interconnected research questions (RQs):

– **RQ1:** What are the current applications and research developments of GAI in sport and exercise science?
– **RQ2:** What major limitations, evidence gaps, and practical challenges are associated with GAI in this field?
– **RQ3:** What ethical issues arise from the application of GAI in sport and exercise settings, and how should future research and practice respond?

By answering these questions, this review provides not only a comprehensive evidence synthesis but also a critical conceptual map to inform future research, practice, and policy in this rapidly evolving domain.

## MATERIALS AND METHODS

This review was conducted and reported in accordance with the Preferred Reporting Items for Systematic Reviews and Meta-Analyses (PRISMA) guidelines for systematic reviews [19], incorporating a rigorous and transparent approach to search strategy development, study selection, quality appraisal, data extraction, and analytic procedures. The review protocol was prospectively registered in the International Prospective Register of Systematic Reviews (PROSPERO; registration number: CRD420251184213).

### Inclusion and Exclusion Criteria

To ensure alignment between the included studies and the objectives of this review, explicit inclusion and exclusion criteria were established based on study focus, research design, publication source, and availability of full-text materials. The specific eligibility criteria applied in this review are summarised in Table 1.

**TABLE 1:** Inclusion and exclusion criteria for study selection.

	Inclusion criteria	Exclusion criterion
Study focus	Studies examining the application of GAI within sport and exercise science domains (e.g., physical education, athlete training, health promotion, exercise rehabilitation). Eligible GAI applications must involve one or more core generative capabilities: Content Generation and Customization: Creating personalized training/rehabilitation plans, exercise instructions, nutritional advice, or motivational feedback. Synthetic Data Generation: Producing simulated human movement data, synthetic imagery for biomechanical analysis, or other artificially generated datasets for research or training. Interactive Generative Systems: Employing LLM-based chatbots, conversational coaches, or agents that generate novel, context-aware responses to user input. Technologies may include systems built on DGMs such as GANs, VAEs, Transformers/LLMs (e.g., GPT, CodeBERT), LDMs, Diffusion Models, or similar architectures whose primary function is the generation of new data instances [20].	Studies that did not involve GAI or were unrelated to sport-, exercise-, or physical activity-related domains. Systems were excluded if they only retrieved, classified, predicted, or reformatted existing information without probabilistic generation of novel data instances, even if natural-language outputs were presented (e.g., traditional machine learning for classification/regression, predictive algorithms in wearables without generative components, rule-based expert systems, retrieval-based dialogue systems, recommender systems without generative capabilities, and purely analytical AI models).

Publication source	Peer-reviewed journal articles or conference papers.	Non–peer-reviewed materials, including opinion pieces, news reports, and book chapters.

Study design	Original research employing experimental designs (e.g., randomised controlled trials, quasi-experimental studies), observational designs (e.g., cross-sectional surveys), or technically oriented application studies with clearly reported methodological procedures.	Secondary research such as bibliometric analyses, systematic reviews, meta-analyses, or scoping reviews.

Availability	Full-text versions accessible for review.	Reports with incomplete information will be excluded. Duplicate or substantially overlapping publications, in which case only the most complete or most recent version was retained.

### Search Strategy

A comprehensive search was conducted by an independent reviewer in four electronic databases—Web of Science, PubMed (including MEDLINE and PubMed Central), SPORTDiscus, and Scopus—using Boolean operators. The search strategy was as follows: (“generative artificial intelligence” OR “generative AI” OR “large language model*” OR “LLM*” OR “ChatGPT” OR “GPT-4” OR “Gemini” OR “Bard” OR “Claude” OR “ERNIE Bot” OR “Sora” OR “Kimi” OR “DeepSeek” OR “Grok” OR “AI-generated content” OR “AIGC” OR “GPT-5”) AND (“sport science” OR “exercise science” OR “kinesiology” OR “physical education” OR “PE” OR “sport training” OR “athletic training” OR “sport performance” OR “rehabilitation” OR “exercise therapy” OR “health promotion” OR “fitness” OR “biomechanics” OR “motor learning” OR “motor control” OR “sport pedagogy” OR “movement analysis”). The complete search strategy is provided in Supplementary Material. Apart from limiting publication types, no restrictions were applied regarding publication year, language, or study setting. The final search was conducted on February 1, 2026. In addition, Google Scholar and ResearchGate were searched using the same search strategy, and the first 30 pages of results were screened, with the screening conducted on February 1, 2026. During this process, each record on every page was assessed through full-text review to identify studies meeting the inclusion criteria. Furthermore, the reference lists of relevant review articles and included studies were manually examined to identify additional eligible publications. All retrieved records were imported into EndNote X9 for reference management and duplicate elimination. Subsequently, titles and abstracts were screened independently against predefined eligibility criteria, followed by full-text assessment of studies deemed potentially relevant. Two reviewers (S.L. and Y.H.) screened independently; disagreements were resolved by a third reviewer (Y.S.). The arbitration procedure is a vote.

### Methodological Quality Assessment

Given the heterogeneity and diversity of study designs, methodological quality was assessed using the suite of appraisal tools developed by the Joanna Briggs Institute (JBI). The JBI instruments provide design-specific checklists (e.g., for cross-sectional studies, cohort studies, case reports, and qualitative research), with each item rated as Yes (= 1), No (= 0), or Unclear/Not applicable (= 0). This structure enables the evaluation of internal validity and reporting quality across diverse study types within a unified framework [21, 22]. The approach has been widely adopted in cross-disciplinary reviews to ensure consistency of appraisal criteria and comparability of results [23]. Although the JBI framework does not prescribe a numerical scoring system, we calculated the percentage of items rated Yes to facilitate descriptive interpretation. Following previous reviews [24, 25], study quality was categorised as low risk of bias (≥ 70%), moderate (50–70%), or high (< 50%). In addition, itemlevel appraisal results were reported to ensure a comprehensive and transparent assessment of methodological quality. Each study was independently appraised by two reviewers (S.L. and Y.H.), and discrepancies were resolved through discussion or adjudication by a third reviewer (Y.S.).

### Data Extraction

Data extraction was performed according to the following variables: author and year, study design, GAI models, prompt structure (information granularity), evaluation source, evaluation focus, intervention or application method, key findings, and risk-of-bias assessment results. Titles, abstracts, and keywords were also collected for use in the LDA topic-modelling analysis. Two independent reviewers (S.L. and Y.H.) conducted data extraction, and any disagreements were resolved through discussion, with arbitration by a third reviewer (Y.S.) where necessary.

### Synthesis of Evidence

#### Bibliometric Analysis

To characterise the research landscape and the evolution of thematic hotspots related to GAI in sport and exercise science, bibliometric visualisations were generated using CiteSpace [26] and SCImago Graphica [27]. First, CiteSpace (version 6.4.R1) was employed to conduct keyword, author, and institutional co-occurrence analyses, as well as keyword clustering, in order to capture the emergence of research frontiers, delineate their developmental trajectories, and identify influential research groups. In CiteSpace, analyses were conducted using annual time slices spanning 2015–2025 (one year per slice). The selection criteria were set to retain the top 50 items per slice based on the highest frequency of occurrence or citation counts. The rest are the default settings of the software.

Subsequently, the resulting .graphml files were exported from CiteSpace and imported into SCImago Graphica (version Beta 1.0.46) to construct geographically oriented collaboration networks. A geographic map layout was applied based on country names, with node sizes scaled according to publication output. Collaborative links were visualized as curved edges, and different countries were distinguished by color. Default layout and rendering parameters were retained, and no additional filtering or pruning was performed in Scimago Graphica. It should be noted that the country-level collaboration analysis is affiliation-based. Given the prevalence of multi-institutional and multinational authorship, a single publication may be assigned to multiple countries, and the resulting networks therefore represent patterns of cross-national co-authorship rather than isolated national contributions.

#### LDA Topic Modeling

To further uncover the latent thematic structure within the included literature, a LDA model was applied to the titles, abstracts, and keywords of all eligible studies. LDA identifies underlying themes by estimating the probability distributions of terms associated with a predefined number of topics, thereby revealing hidden semantic patterns within a document corpus [28, 29]. Prior to topic modelling, bibliographic records were pre-processed by merging titles, abstracts, and keywords, followed by text normalization (lowercasing, removal of non-alphabetic characters), length-based token filtering, and domain-specific stopword exclusion. A bag-of-words representation with uni- and bi-grams was constructed using document-frequency thresholds (min_df = 2, max_df = 0.4) to reduce noise and overly generic terms, ensuring a stable and semantically discriminative input for LDA (see Supplementary Material for specific operational code). LDA modelling was implemented in Python (version 3.13.5). To determine the optimal number of topics (K), both perplexity and coherence metrics were evaluated [30], supplemented by iterative testing informed by domain knowledge. Each topic was represented by a set of high-weight keywords, and topic labels were assigned by the research team based on semantic interpretation and alignment with the corresponding literature. To enhance interpretive reliability, two researchers (S.L. and Y.H.) independently reviewed and refined topic labels through discussion, ensuring conceptual coherence and reproducibility [31]. The resulting LDA-derived thematic framework informed the subsequent synthesis and interpretation of evidence. Figure 1 presents the conceptual model of the LDA topic-modelling approach used in the present study, and its probabilistic formulation is shown below:

**FIG. 1:**
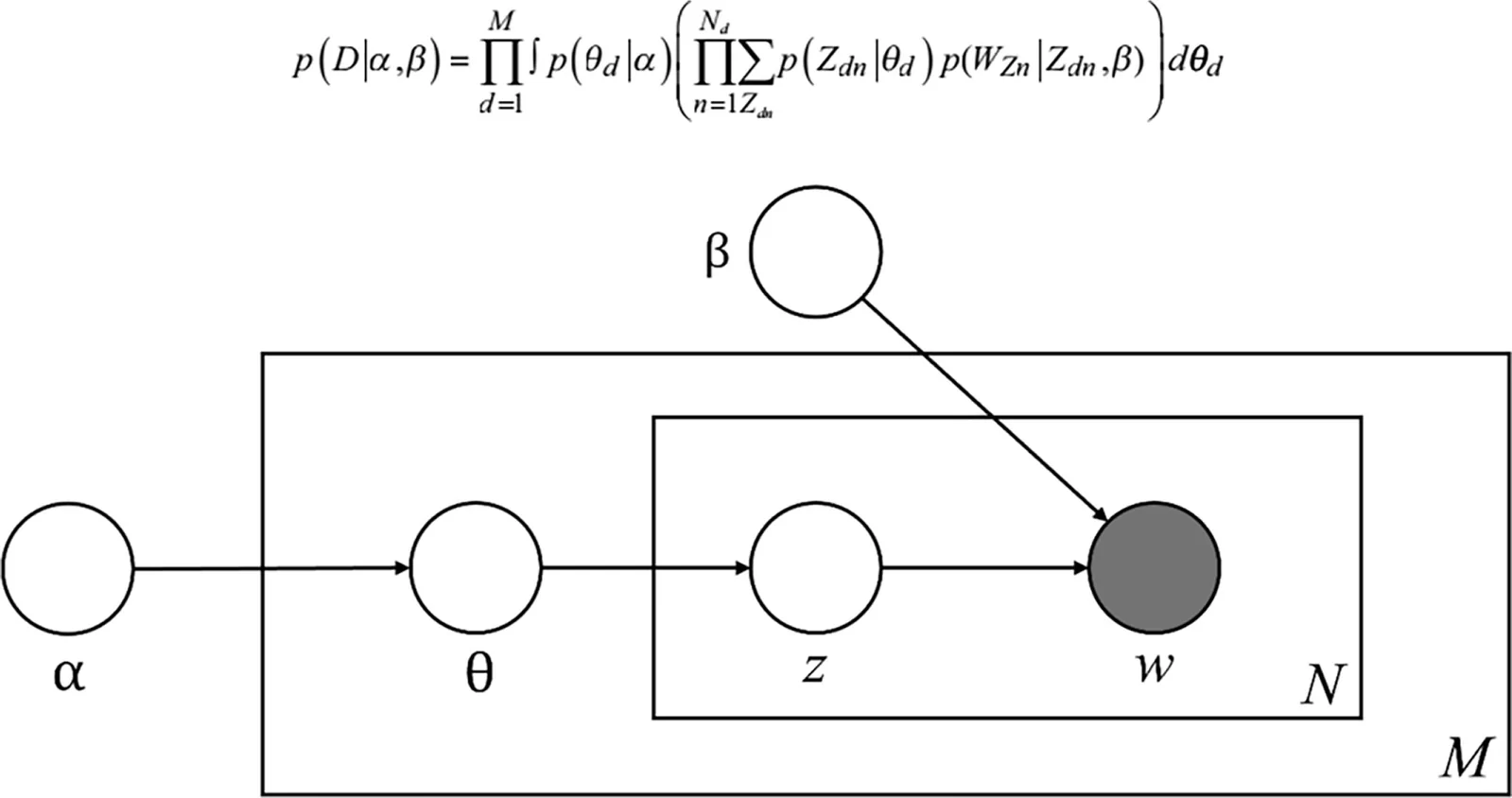
Probabilistic graphical model of LDA. α: a parameter that represents the Dirichlet prior for the document topic distribution, β: a parameter that represents the Dirichlet for the word distribution, θ: a vector for topic distribution over a document d, z: a topic for a chosen word in a document, w: specific words in N, M: the length of documents, N: the number of words in the document.

#### Research Description

A narrative synthesis approach was employed to integrate and summarise the findings descriptively, allowing the identification of commonalities and divergences across studies [32]. To enhance the readability and practical applicability of the research findings, a following coding strategy was adopted. Two reviewers (S.L. and Y.H.) independently coded all included studies into one of the four narrative subdomains (physical education and pedagogical innovation, personalised training and fitness programs, exercise rehabilitation and clinical prescription, and health promotion and behaviour change) using predefined decision rules: (1) primary research objective, (2) target population, and (3) application context. Disagreements were resolved through discussion, with adjudication by a third reviewer (Y.S.) when necessary.

## RESULTS

## Research Characteristics

The study selection process is summarised in the PRISMA flow diagram (Figure 2). A total of 1518 records were initially identified through database searches. Following independent screening by two reviewers 47 studies met the eligibility criteria, with an additional 17 studies identified through supplementary sources. Ultimately, 64 studies were included in the final review [10–14, 33–91].

**FIG. 2:**
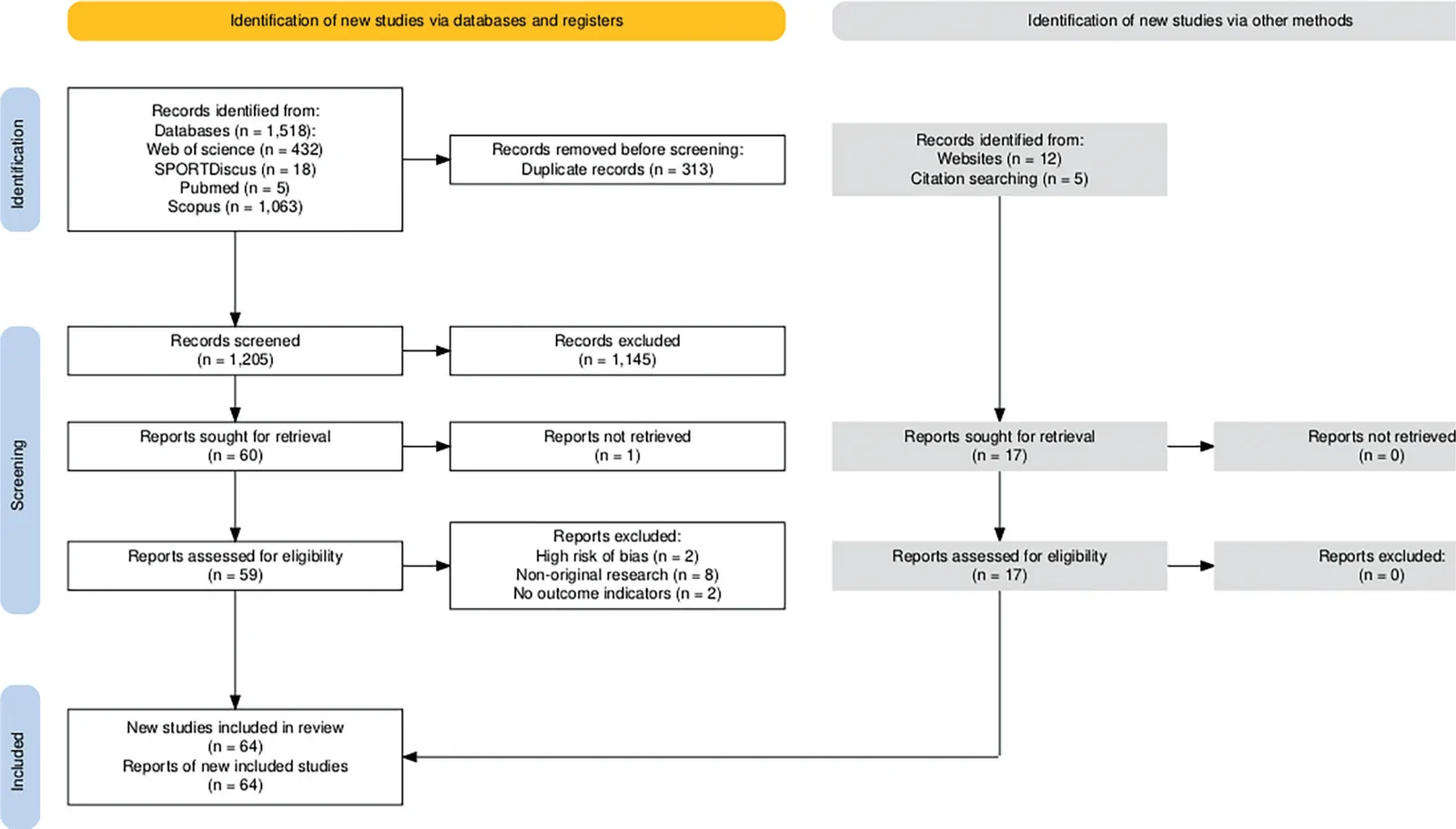
Flow diagram of article retrieval and screening.

All study characteristics were independently extracted by two reviewers, and no disagreements arose during the process.Full dataset is provided in Supplementary Material. Given the methodological differences between artificial intelligence research and traditional epidemiological study designs [92], and to facilitate quality quantification or descriptive reporting, the 64 included studies were classified according to the following research design categories: 2 employed RCT, 5 adopted quasi-experimental designs (causal research involving deliberate ‘non-randomised’ interventions), 31 used cross-sectional methods, 7 were qualitative investigations, and 19 were system development and validation studies. With respect to AI models, ChatGPT (versions 3, 3.5, 4, 4o, 4o-mini, o3, and o3-mini) was most frequently used, followed by Sora, Google Gemini, DeepSeek (R1, V3), as well as customised chatbots built upon LLMs, GANs or VAEs. In terms of application domains, 11 studies focused on physical education, 23 on health promotion, 13 on exercise rehabilitation, and 17 on sport training. Across these domains, GAI was primarily applied to training- and teaching-content generation, intelligent health assessment and feedback, and personalised health-management support. The evaluation populations for GAI included end users such as coaches, domain experts, clinicians, and physiotherapists, with primary assessment focuses on accuracy, effectiveness, and safety.

## Quality of Research

This study employed JBI appraisal tools to quantitatively assess the risk of bias in 45 included studies, while an additional 19 system development and validation studies were evaluated descriptively due to the absence of suitable scoring instruments.

Among the quantitatively appraised studies, overall quality scores—calculated as the proportion of items rated “Yes”—ranged from 60% to 100% (mean: 81%), with detailed item-level ratings reported in Supplementary Material. Of these studies, 37 (82%) were classified as low risk of bias and 8 (18%) as moderate risk; no studies were rated as high risk, indicating generally acceptable methodological quality.

Across study designs, the two RCTs exhibited substantial limitations in allocation concealment and blinding (both rated “No” in 100% of cases) (Figure 3). Quasi-experimental studies (n = 5) showed adequate performance in temporal sequencing, pre–post design, and outcome measurement consistency, but insufficient reporting of control group use and baseline comparability (40% and 25% rated “No,” respectively), suggesting residual selection bias (Figure 4). Cross-sectional studies (n = 31) generally demonstrated high reporting compliance, particularly for inclusion criteria, measurement validity and reliability, and statistical analyses; however, confounding control was frequently inadequate, with only 50% identifying potential confounders and 23% reporting strategies to address them (Figure 5). Qualitative studies showed high coherence between research questions, methodology, data collection, analysis, and conclusions (typically 86–100% “Yes”), but performed poorly in philosophical–methodological alignment and researcher reflexivity (29% “Yes”) (Figure 6).

**FIG. 3:**
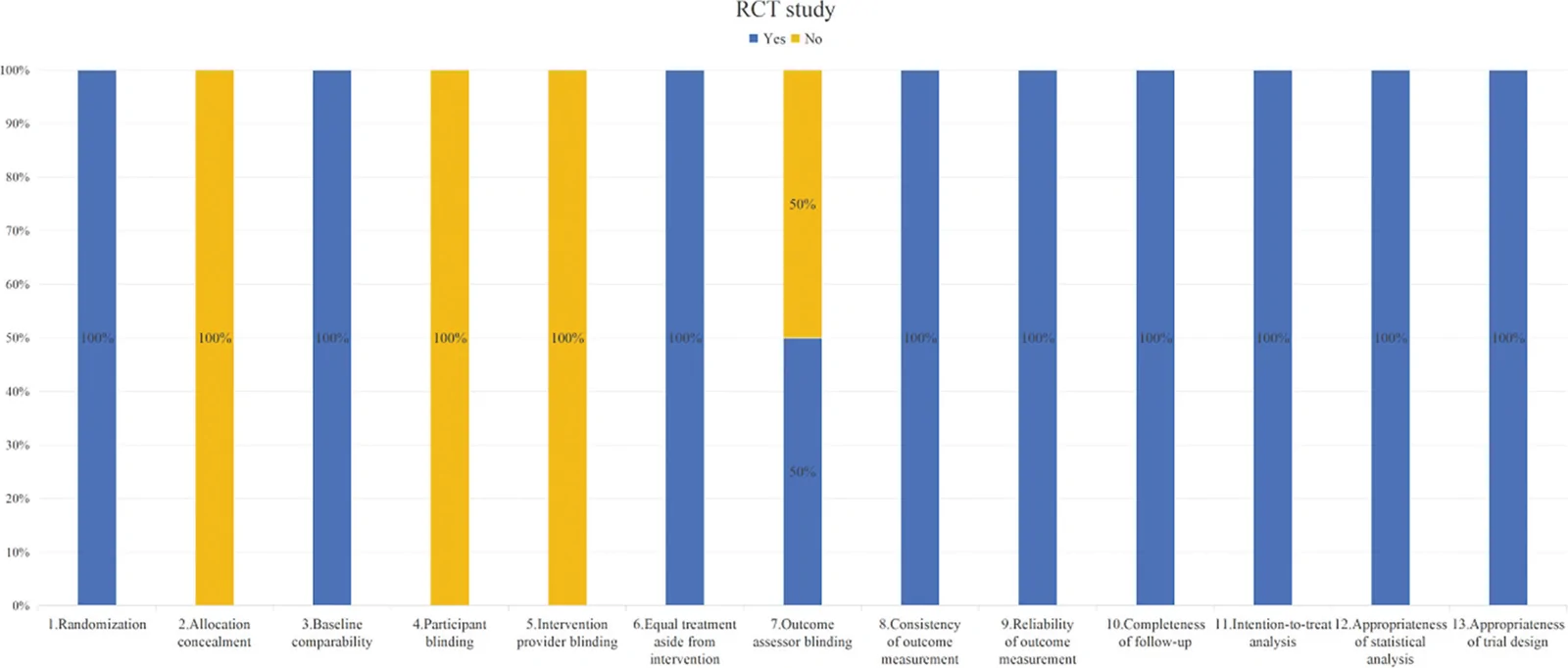
Item-level methodological quality profile of RCTs (Generated by Excel).

**FIG. 4:**
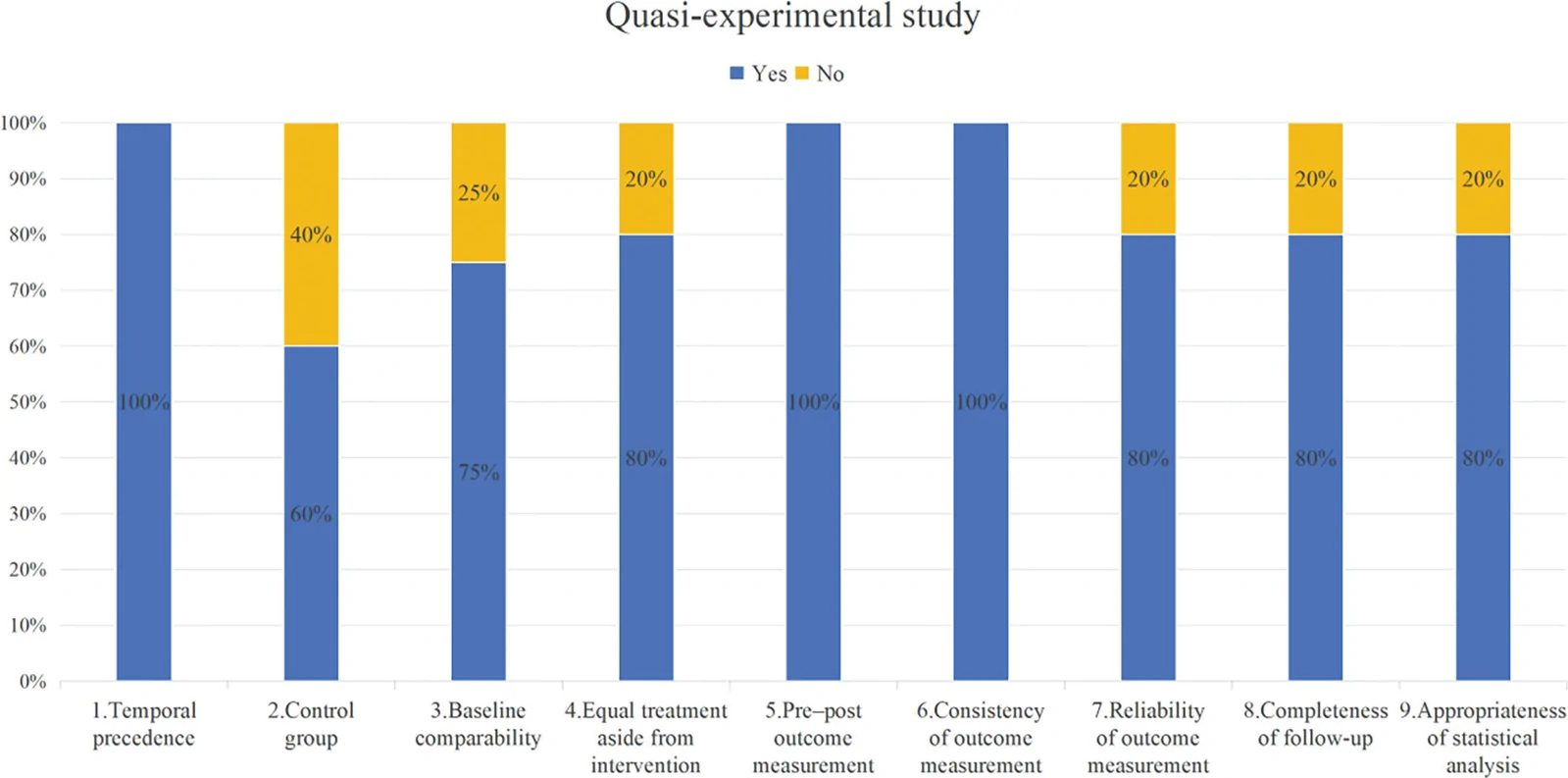
Item-level risk-of-bias profile of quasi-experimental studies (Generated by Excel).

**FIG. 5:**
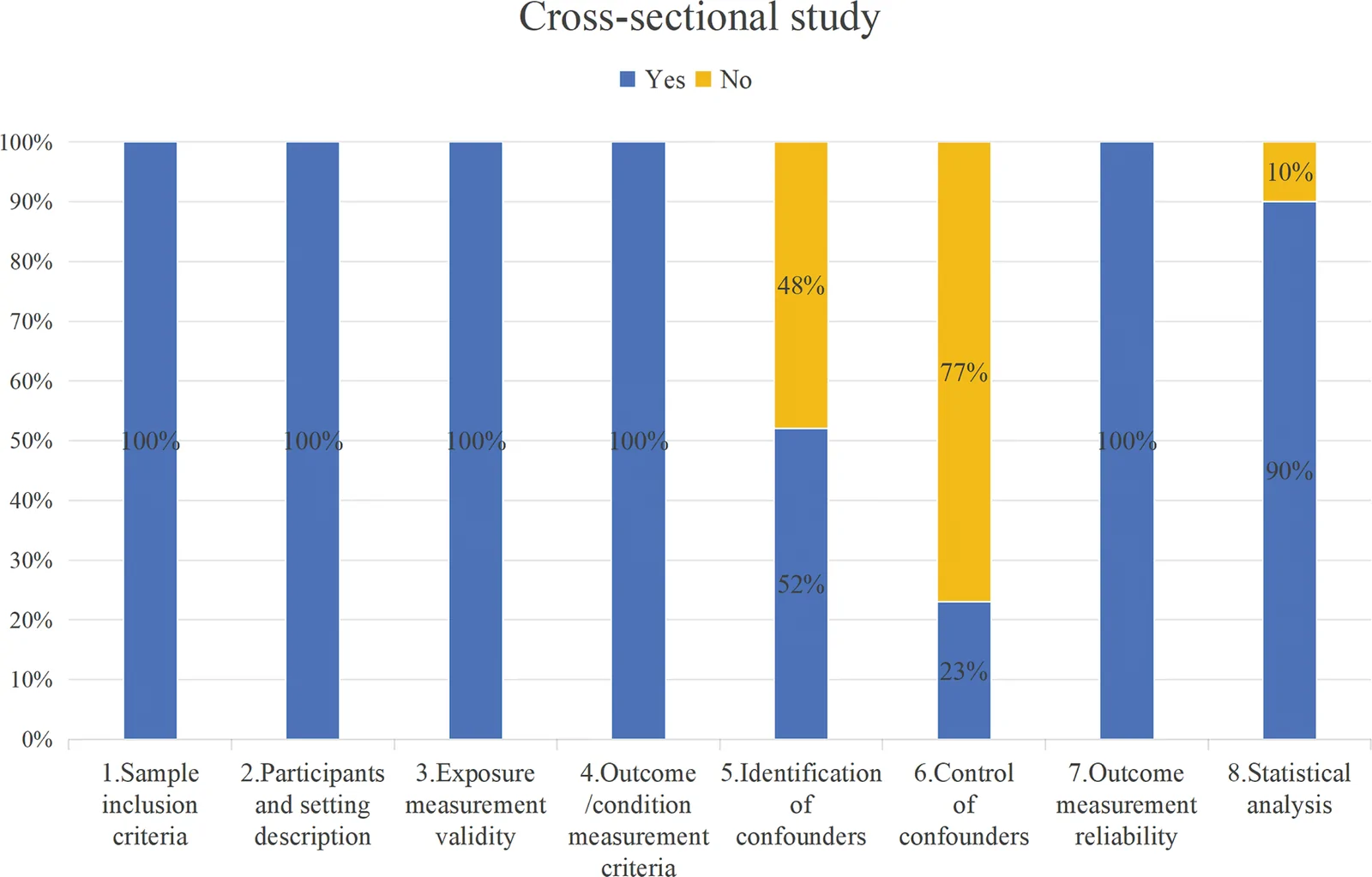
Item-level methodological quality distribution of cross-sectional studies (Generated by Excel).

**FIG. 6:**
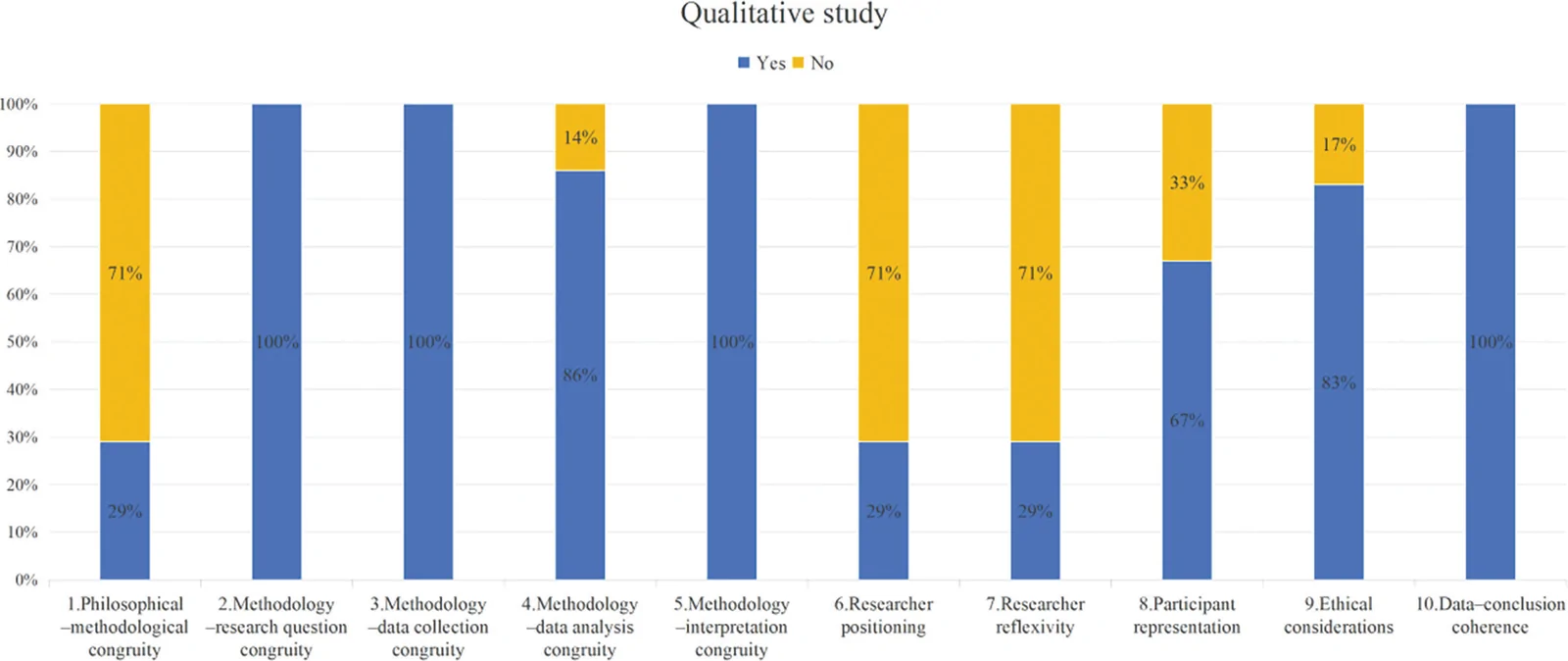
Item-level methodological rigor profile of qualitative studies (Generated by Excel).

The 19 system development and validation studies demonstrated adequate technical validity and internal logical consistency, with most clearly reporting development procedures, validation processes, and performance evaluations. Nevertheless, common limitations included the absence of real-world implementation, limited external validation, and insufficient user-centered or contextual evaluation.

Overall, while the included literature exhibited acceptable methodological rigor, bias structures varied substantially across study designs. In particular, the evidential strength of system development and validation studies remains constrained by limited external generalizability and real-world validation, warranting cautious interpretation when formulating practice-oriented recommendations.

## Bibliometric Analysis

### Collaboration Networks of Countries, Institutions, and Authors

Figure 7 illustrates collaboration networks at the national, institutional, and author levels. At the national level, research on generative AI in sport and exercise science shows a geographically distributed but weakly connected pattern, led by China (17 studies), the United States (14 studies), and Turkey (8 studies). Despite their relatively higher publication output, overall productivity remains modest, and cross-national collaboration is still at an early stage, characterised primarily by intra-regional links and a diffuse global structure without dense international networks (Figure 7b).

**FIG. 7:**
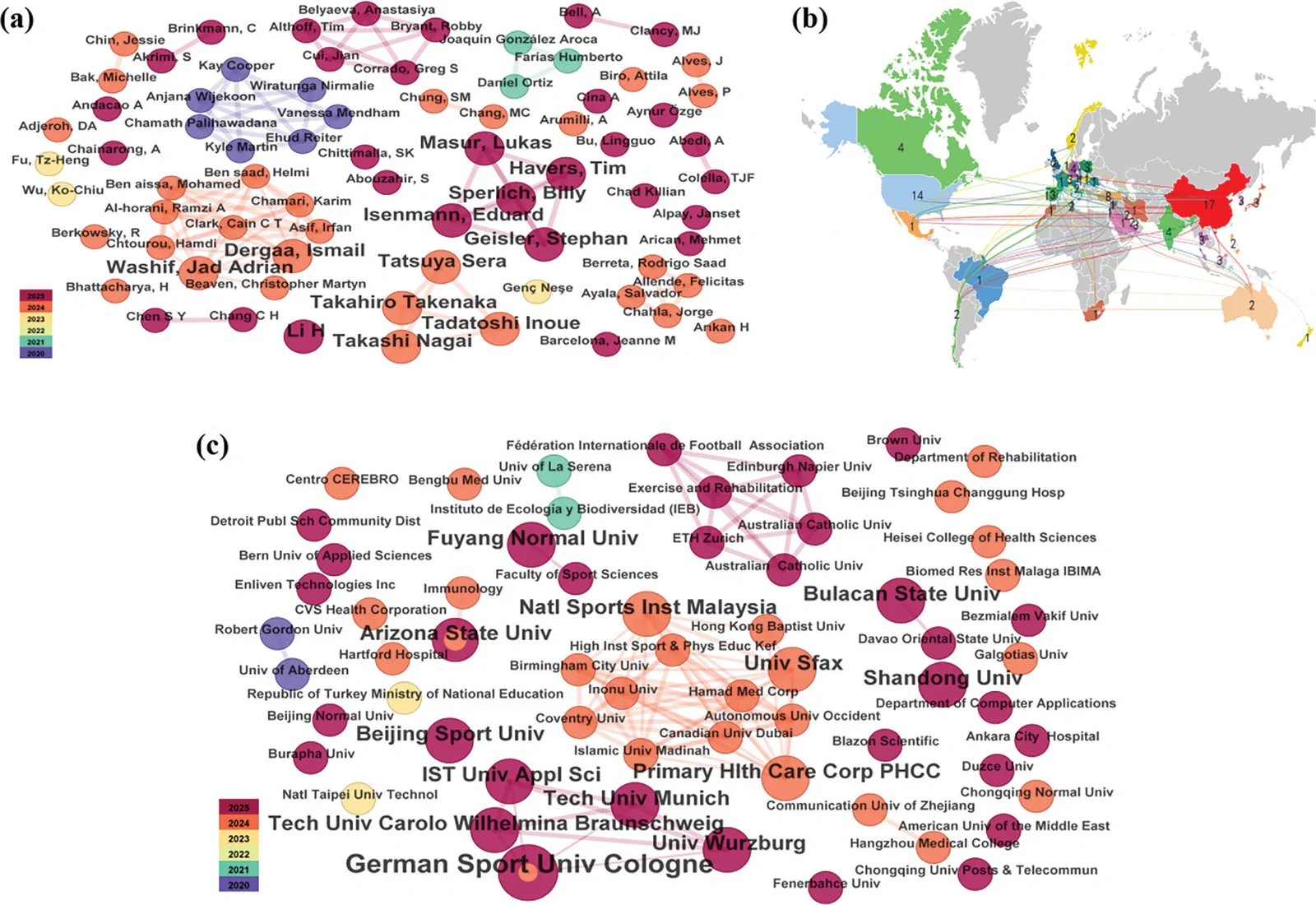
Visualization of bibliometric networks (Generated by CiteSpace 6.4.R1): (a) Co-authorship; (b) National/Regional collaboration; (c) Institutional co-occurrence.

At the institutional level (Figure 7c), several preliminary core research units have emerged. Institutions such as Stanford University and German Sport University Cologne appear as key nodes with comparatively higher output. However, inter-institutional collaboration remains limited, with no large-scale, multi-institutional or cross-regional clusters identified, suggesting a dispersed research capacity.

The co-authorship network (Figure 7a) further indicates fragmentation within the research community. Only a small number of author groups demonstrate close collaboration, while connections between clusters remain sparse. Overall, these network patterns reflect the nascent stage of GAI research in sport and exercise science, in which exploratory studies are largely conducted independently within specialised subfields.

### Distributional Characteristics of Keywords

The keyword co-occurrence analysis (Figure 8a) revealed several concentrated areas of research activity. High-frequency terms such as “artificial intelligence,” “generative AI,” “exercise prescription,” “physical activity,” “information use,” and “digital health” reflect the primary application domains of generative AI across physical education, training, health management, and rehabilitation. In addition, technical terms including “large language model,” “natural language processing,” “app design” and “ChatGPT” appeared frequently, indicating that scholarly attention extends not only to application contexts but also to specific AI architectures and tools. Keyword clustering analysis (Figure 8b) further delineated thematic structures, revealing distinct clusters associated with sports medicine, physical education, and digital health. Collectively, these results suggest that research hotspots in this field are becoming increasingly well defined. Current focal areas include intelligent instructional design, personalised training optimisation, digitalised health management, and AI-assisted rehabilitation. Moreover, the clustering patterns demonstrate that these themes continue to expand and refine in parallel with advances in generative AI technologies.

**FIG. 8:**
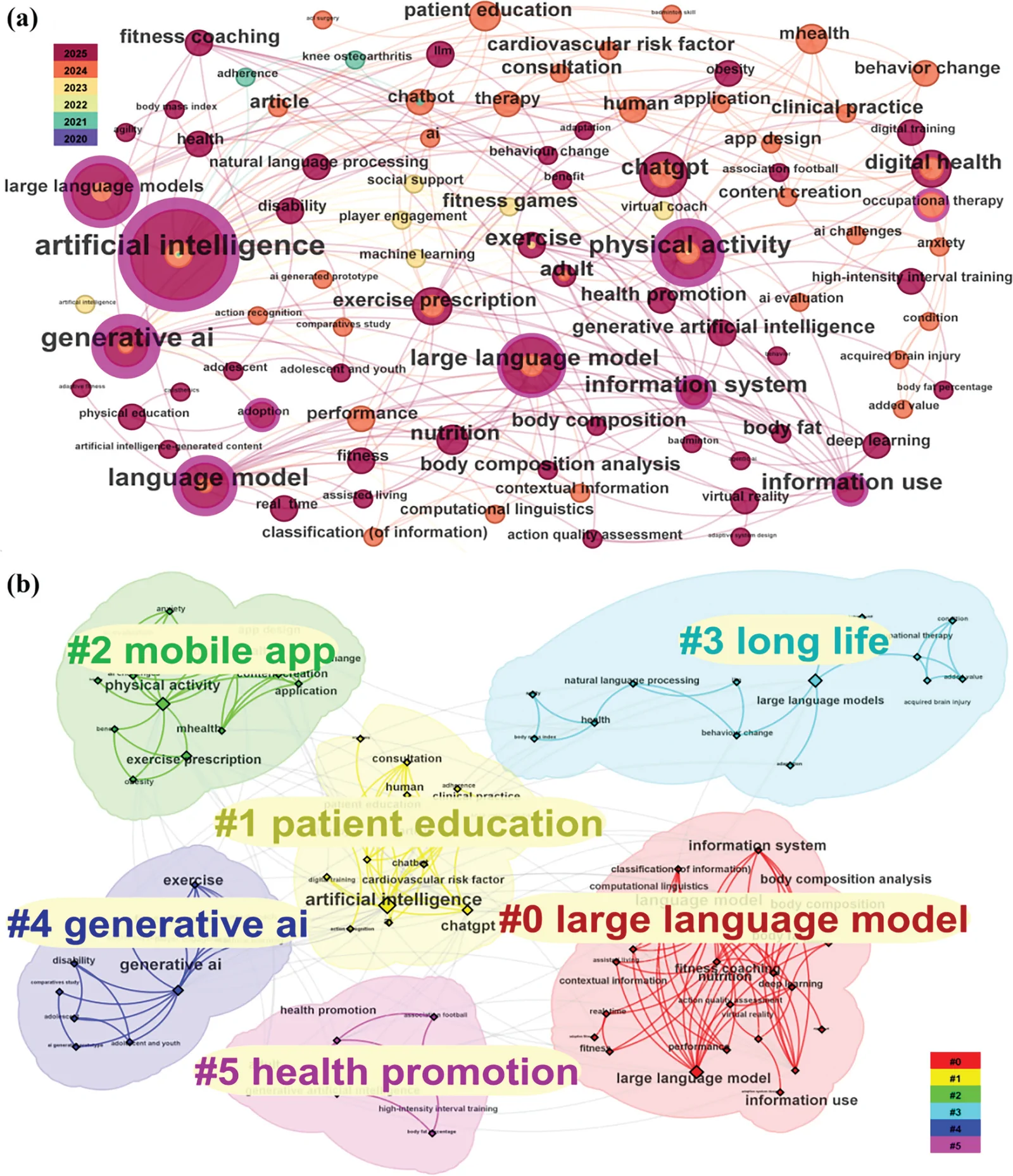
Keyword distribution feature network (Generated by CiteSpace 6.4.R1): (a) Keyword co-occurrence; (b) Keyword clustering.

## LDA Topic Modeling

To determine the optimal number of topics (K), model scores, perplexity values, and coherence indices were calculated for models with K ranging from 2 to 8. Iterative testing revealed that the model achieved its highest score at K = 2, accompanied by the highest coherence and lowest perplexity values (Figure 9a,b). These results indicate that the two-topic solution provided the best balance between topic distinctiveness and model stability. Accordingly, K = 2 was selected as the optimal solution, and the top 15 keywords for each topic were used for thematic interpretation and visualisation (Table 2).

**FIG. 9:**
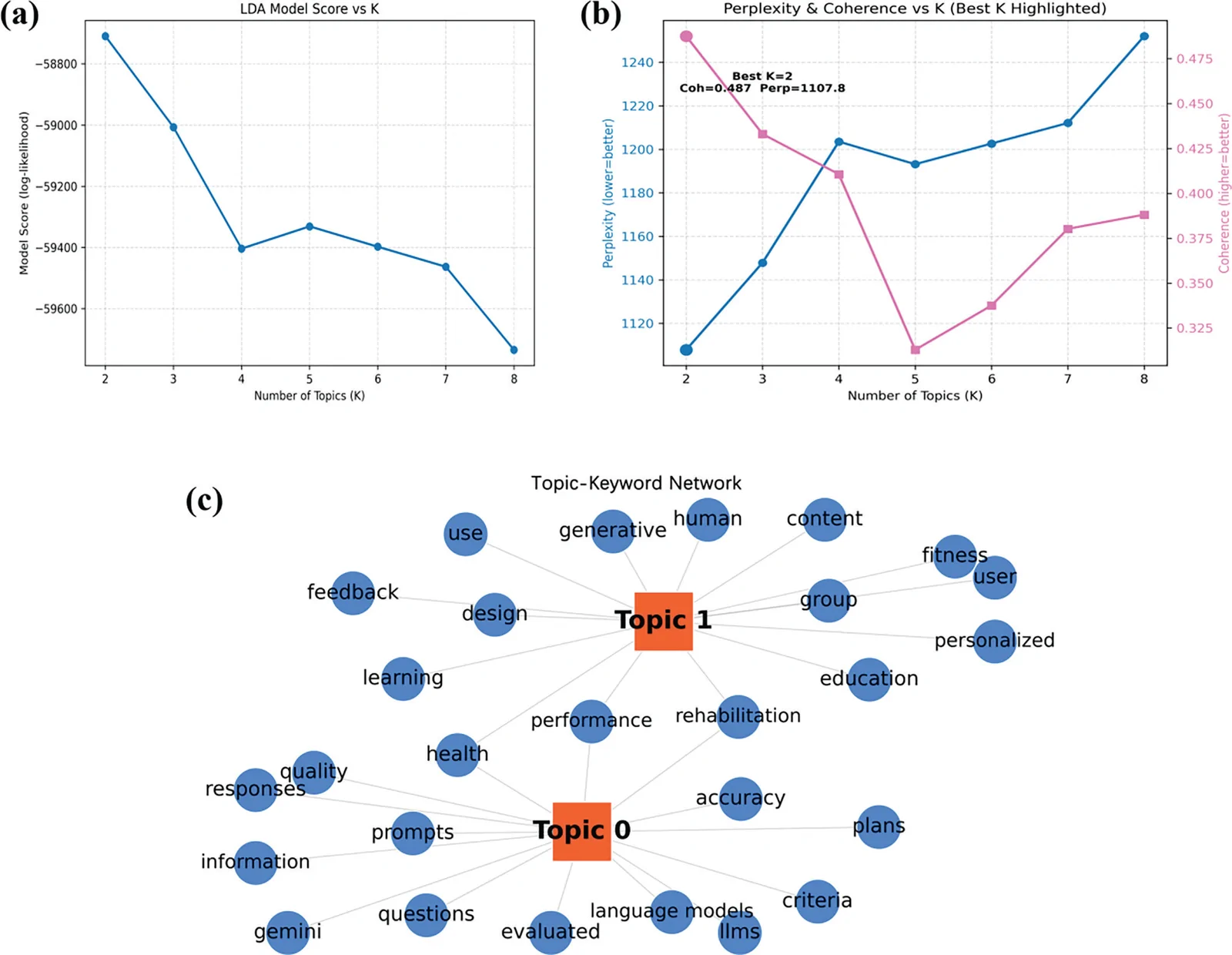
LDA topic modeling results (Generated by Python 3.13.5): (a) The model scores under different themes; (b) Perplexity and coherence of models under different themes; (c) Topic-keyword semantic network.

**TABLE 2:** The result of LDA topic modeling.

	Topic 0	Topic 1

Semantics	Methodological evaluation of GAI-generated content in sport, health, and rehabilitation contexts	Application-oriented research on personalised GAI systems in sport, health, and education

Keywords	plans	fitness
	questions	learning
	information	health
	accuracy	rehabilitation
	quality	personalized
	rehabilitation	use
	performance	generative
	responses	performance
	language models	design
	llms	group
	gemini	education
	prompts	user
	health	content
	evaluated	human
	criteria	feedback

As shown in Table 2, the LDA model identified two principal topics with distinct semantic structures. Topic 1 represents an evaluation-oriented research stream focused on the generation and assessment of GAI outputs in sport, health, and rehabilitation contexts. High-weight keywords (e.g., plans, accuracy, quality, criteria, responses), together with technical terms related to large language models and prompting (e.g., LLMs, prompts, Gemini), indicate that this topic primarily examines the scientific validity, methodological reliability, and performance of GAI-generated content, rather than its direct real-world implementation.

In contrast, Topic 2 reflects an application-oriented research stream centred on the practical deployment of generative AI across fitness, physical education, health, and rehabilitation settings. Keywords such as fitness, education, personalized, feedback, learning, and user highlight a focus on individualised interventions, human–AI interaction, and user-centred design, with studies investigating how GAI systems are integrated into real-world training, teaching, healthpromotion, and rehabilitation scenarios.

Taken together, these two topics distinguish between the methodological evaluation of GAI-generated content and its applied use in sport and exercise science. The corresponding keyword networks are shown in Figure 9c.

## Research Description

Overall, quantitative analyses based on keyword co-occurrence, clustering, and LDA topic modelling delineated the application landscape of GAI in sport and exercise science. At a latent semantic level, these approaches consistently identified two overarching trajectories: (i) evaluation and quality assessment of GAI-generated content, and (ii) application of GAI in real-world contexts, including training, education, rehabilitation, and health promotion.

To improve practical interpretability, the descriptive synthesis was reorganised by application context rather than model-derived topics. Accordingly, the evidence was structured into four subdomains: physical education and pedagogical innovation; personalised training and fitness programmes; exercise rehabilitation and clinical prescription; and health promotion and behaviour change. Within each subdomain, studies were synthesised using an application pathway–oriented framework encompassing three progressive categories: (1) evaluation of GAI-generated content, (2) practical application and intervention, and (3) development of domain-specific or specialised GAI systems. This framework reflects increasing levels of GAI integration and enables structured comparison of research objectives, evaluation approaches, and evidential strength across studies. Detailed descriptions are provided below.

### Physical Education and Pedagogical Innovation

In physical education research, only three studies evaluated the quality, accuracy, and pedagogical applicability of GAI-generated content rather than its direct effects on teaching or learning outcomes. These studies suggest that ChatGPT achieves relatively high accuracy and usability in instructional resource generation, curricular support, and sport-related cognitive assessment, with potential benefits for teaching efficiency and inclusivity [33, 34, 42]. However, accuracy risks, over-reliance, and ethical concerns remain, and empirical evidence of instructional effectiveness is still limited [34, 42].

Regarding practical applications, experimental studies generally report positive effects of GAI-assisted instruction on learning efficiency, learner engagement, and overall learning outcomes [39, 41]. Evidence for motor skill acquisition, however, is inconsistent and may indicate adverse effects when expert supervision is insufficient [41]. Questionnaire-based studies further show that integrating GAI into physical education enhances perceived usefulness, engagement, intention to use, and perceptions of inclusivity [36–38, 43], while persistent concerns about accuracy, ethics, data privacy, and over-reliance continue to constrain wider adoption [36, 37, 43].

At the system-development level, Ma et al. validated a table-tennis instructional system integrating a multimodal large language model (GPT-4) with a domain-specific knowledge base in authentic teaching contexts. The system demonstrated acceptable ecological validity for error detection and feedback accuracy (mean accuracy = 0.67), indicating potential cost-effectiveness for novice learners [35].

### Personalized Training and Fitness Programs

The ten included studies on sport training primarily examined the accuracy, consistency, personalisation, and safety of GAI-generated training programmes. Under semi-structured or FITT-based structured prompts, programmes generated by ChatGPT-4/4.5 and Gemini generally demonstrated moderate to good performance in overall structure, periodisation logic, and guideline adherence [10, 11, 46–49, 53, 54], with some studies reporting quality comparable to, or difficult to distinguish from, human-designed programmes [53]. However, recurrent limitations were identified in goal-specific differentiation (e.g. hypertrophy vs. strength), prescription of moderate-to-high intensity zones, energy system distribution, and individual health screening, necessitating frequent manual revision [47–49, 54]. Prompt information granularity emerged as a key determinant of output quality, as highinformation and structured inputs substantially improved programme quality, stability, and reproducibility [11, 46, 50]. User-level studies further indicated high acceptance and trust among athletes, although evidence supporting independent AI-based training decision-making remains insufficient [44, 53].

Evidence from three quasi-experimental studies suggests that GAIgenerated training programmes can improve flexibility, muscular endurance, strength, cardiorespiratory fitness, and psychological wellbeing in applied settings [45, 51, 52]. Nevertheless, compared with human-coach-designed programmes, GAI-based plans were less effective for complex objectives such as sport-specific endurance and bilateral explosive power, and further validation is required regarding effect stability and depth of individualisation [51].

Four system development and validation studies demonstrated that GAI-enabled systems can support training plan generation, movement error detection, and real-time feedback through structured prompting, retrieval-augmented generation, multi-agent architectures, and multimodal sensing. These systems showed generally good usability, guideline compliance, and analytical accuracy, providing coach-level or near coach-level support [55, 58]. However, their evidential strength remains limited by prototype-level validation, reliance on visual data quality, constrained deep personalisation, and the absence of longterm effectiveness evaluation [55–57].

### Exercise Rehabilitation and Clinical Prescription

Eight evaluative studies in the exercise rehabilitation domain primarily assessed the accuracy, guideline adherence, personalisation, and clinical appropriateness of GAI-generated content. Under structured, high–information-granularity prompts, responses generated by ChatGPT-4/4o and Gemini for rehabilitation scenarios such as anterior cruciate ligament reconstruction, osteoarthritis, and common musculoskeletal conditions generally achieved moderate to high accuracy and guideline consistency, performing well in the provision of general rehabilitation knowledge and educational support [12, 13, 59–61, 64]. ChatGPT-4 consistently outperformed ChatGPT-3 [64]. However, recurrent limitations were reported in dosage prescription, progression logic, clinical reasoning, and complex decision-making, alongside instability in emotion recognition and privacy risk management [12, 61, 62]. In addition, GAI systems demonstrated the capacity to generate personalised, emotionally engaging, game-based narratives tailored to individual patient characteristics [14].

System development and validation studies mainly applied GAI to automate rehabilitation assessment and feedback using multimodal sensing (e.g. motion skeletons, biomechanical data, virtual reality), structured prompting, and fine-tuned language models. These systems showed strong performance in movement quality assessment accuracy, feedback consistency, and interpretability, with reported performance comparable to, or approaching, deep learning baselines, and advantages in personalised recommendations and professional-level communication [63, 66, 68]. Positive effects were also observed for usability, user acceptance, and behavioural intention [63, 65]. Nevertheless, challenges remain regarding stability, reproducibility, and overconfidence in complex datasets, and the current evidence base is largely limited to validation studies, with a lack of long-term clinical outcome evaluation [67].

### Health Promotion and Behaviour Change

Thirteen studies evaluated the performance of GAI—predominantly ChatGPT-3.5/4/4o—in public health–related content generation, focusing on accuracy, safety, personalisation, and usability. Under structured or fixed Q&A prompts, GAI-generated exercise prescriptions, health advice, and behaviour-change interventions generally demonstrated good guideline adherence, overall accuracy, and generation efficiency [69–73, 77–80, 82, 83], and performed well in exercise log classification tasks [89], supporting their potential role as decision-support tools. However, recurrent limitations were identified in personalisation, comprehensiveness, and safety risk identification, particularly for individuals with chronic comorbidities, functional impairments, or clinically complex conditions, where outputs were often generic, incomplete, or required expert review [69–71, 73, 77–79, 82, 83]. In behaviour-change applications, GAI showed better performance in identifying action and maintenance stages than in effectively addressing precontemplation or contemplation stages [90]. Although high-information and structured prompting improved output quality, substantial variability across models and prompts persisted, and fundamental challenges related to safety and contextual adaptation remained unresolved [73, 78–80].

Evidence from a randomised controlled trial indicated that an 8-week exercise intervention generated by ChatGPT-4 significantly improved BMI, post-exercise heart rate, and muscular endurance in overweight young adults; however, these findings should be interpreted cautiously due to a moderate risk of bias [75].

Nine system development and validation studies examined GAIbased systems supporting personalised exercise and health management through multimodal integration, wearable data, and structured prompting. These systems generally demonstrated good usability, personalised feedback accuracy, and user engagement, with some achieving or approaching expert-level performance in tasks such as posture recognition, sleep analysis, dietary assessment, and fitness guidance [74, 81, 84, 86–88]. Nonetheless, persistent limitations were reported in deep personalisation, long-term interaction stability, and contextual understanding, often resulting in generic or inconsistent recommendations [81, 85]. While structured prompts, retrieval-augmented generation, and multi-source data fusion improved output consistency and health relevance [76, 88], most systems remain at the prototype or usability-validation stage, and their longterm effectiveness in health promotion requires confirmation in realworld settings [76, 91].

## DISCUSSION

This systematic review, based on 64 studies, addresses three research questions concerning the applications, limitations, and ethical implications of GAI in sport and exercise science.

Regarding RQ1, the findings indicate a rapid expansion of GAI applications across domains such as physical education, personalised training, exercise rehabilitation, and health promotion. Overall, the field is transitioning from general-purpose text generation toward more domain-specific and application-oriented uses, although most studies remain evaluative or exploratory rather than intervention-driven. With respect to RQ2, the methodological quality of the included studies was generally acceptable (mean JBI score: 81%), yet important limitations persist, including a lack of high-quality randomised controlled trials, small sample sizes, short intervention durations, and limited real-world validation. In relation to RQ3, the review highlights emerging ethical challenges associated with the use of conversational GAI tools, particularly in relation to data privacy and governance, algorithmic bias, informed consent, user autonomy, and accountability.

The following sections elaborate on these findings by discussing current applications (RQ1), key limitations and research gaps (RQ2), and ethical challenges and future directions (RQ3) in greater detail.

### Current Applications and Research Progress of GAI in Sport Science (RQ1)

Synthesising the available evidence reveals a vertically evolving trajectory of GAI integration in sport and exercise science, progressing from problem understanding and content generation to practical application and, more recently, toward integrated system-level innovation.

In its early applications, GAI primarily functioned as a consultative tool for interpreting sport-related problems. Large language models were used for expert-level question answering, consolidation of sports medicine knowledge, and interpretation of technical movements, enabling rapid access to structured information for coaches, athletes, and patients [49, 59, 69, 71, 80]. At this stage, research largely focused on usability demonstration, with technical capabilities mainly limited to LLM-based knowledge repositories and natural language understanding and generation.

As analytical capabilities advanced, GAI systems increasingly generated exercise prescriptions and training plans tailored to individual needs. Under structured, high–information-granularity prompting, studies across physical education, training, rehabilitation, and health management consistently reported favourable performance in content quality, structural coherence, guideline adherence, and crosscontext applicability [10–13, 42, 46–48, 53, 54, 60, 64, 70–73, 77–79, 82, 83], broadly aligning with findings from medical research [93]. However, this evidence is derived almost exclusively from content-level assessments and expert appraisals, limiting conclusions regarding real-world effectiveness. From a methodological perspective, most studies relied on cross-sectional expert appraisal. Although such designs have a degree of scientific validity for preliminary evaluation [94], many studies failed to adequately identify or control for potential confounding factors, thereby limiting the strength of causal inference.

In addition, prompt engineering emerged as a key determinant of output quality, with detailed and constrained prompts producing more stable and higher-quality outputs [47, 50, 72]. Nevertheless, repeated generations under identical prompts often yielded variable structures [11], reflecting persistent limitations in controllability. Importantly, improvements in generation quality have not been shown to translate into superior adherence or training and rehabilitation outcomes in real populations. From an evidence-hierarchy perspective, most current applications therefore remain at a pre-validation stage, addressing whether GAI can generate content and whether that content is of acceptable quality, rather than whether it improves outcomes.

More recent studies have begun deploying GAI in real-world teaching, training, and rehabilitation contexts. In physical education, AIassisted instruction has been associated with improved learning efficiency and engagement [39–41]. In sport training, GAI-generated programmes have demonstrated gains in strength, muscular endurance, agility, and cardiorespiratory fitness [45, 52]. In health management, an 8-week exercise intervention generated by ChatGPT-4 improved BMI, post-exercise heart rate, and muscular endurance in overweight young adults [75]. However, these findings should be interpreted cautiously, as existing randomised and quasi-experimental studies exhibit notable design limitations and moderate risk of bias, and challenges such as hallucinations and insufficient personalisation persist [41, 51].

To enhance the applicability of GAI in sport practice, an increasing number of studies have adopted multimodal, cross-system, and multi-agent approaches. Systems integrating domain knowledge and multimodal sensing achieved moderate error-detection accuracy in table-tennis instruction [35], while rehabilitation systems using intelligent wearables enabled real-time task adaptation and personalised feedback [63, 66]. In health management, PH-LLMs combining wearable data and real-time monitoring achieved expert-level or near expert-level performance in sleep and fitness assessment tasks [84]. Multi-agent collaboration and cross-modal reasoning further support complex-task coordination and adaptive decision-making [58]. Despite these advances, most systems have been evaluated primarily for usability during early development, with limited long-term validation in real-world populations.

Overall, GAI applications in sport and exercise science have progressed from problem interpretation to content generation, practical deployment, and emerging system-level integration. This trajectory reflects a shift from assistive use toward partial decision-support. Nevertheless, challenges related to domain specificity, controllability, safety, and validation remain unresolved, underscoring the need for more rigorous, long-term experimental research.

### Primary Limitations, Research Gaps, and Practical Challenges in the Application of GAI in Sport Science (RQ2)

Despite the promising outlook for GAI in sport and exercise science, this review also identified several substantive limitations and challenges that constrain its current utility. At the research level, both the quantity and quality of existing studies remain limited. Most studies employed small samples, short intervention periods, or pilot designs, with few randomised controlled trials, long-term follow-ups, or multicentre investigations—substantially restricting external validity. Many studies evaluated only single-instance model outputs and did not examine output stability, test–retest reliability, or dynamic intervention effects. Although bias appraisal indicated generally moderate-to-good methodological quality, common issues persisted, including inadequate control measures, absence of blinding, and inconsistency in outcome assessment. Consequently, the evidence base remains preliminary and exploratory.

A recurring concern across domains is that while GAI-generated recommendations are often directionally sound, they lack the granular precision required for sport-science practice. In training contexts, AI-generated plans generally adhere to basic training principles but perform poorly in individualisation, sport specificity, and progressive overload compared with coach-designed programmes [51, 52]. In motor-learning scenarios, hallucinations in LLM outputs may lead to incorrect technique descriptions, potentially causing movement errors or injury risk [41]. For health advice and exercise prescriptions, considerable variability exists between models, and AI often lacks sufficient safety boundaries or individualised adjustments for high-risk populations such as older adults or individuals with chronic disease. These findings highlight a persistent limitation: GAI tends to offer “broadly correct but insufficiently precise” advice. Improving accuracy, consistency, and individualisation remains a key technical bottleneck.

Practical adoption of GAI in sport science is further complicated by human- and context-related constraints. User acceptance and trust represent major barriers. Teachers express concerns about reduced deep learning and academic integrity [95]; coaches view AI as more suited for novices and insufficiently capable of handling complex sport-specific scenarios [53]; rehabilitation practitioners worry about inadequate safety oversight for vulnerable patients [78]. Such reservations may impede the diffusion of GAI tools in professional sport settings. Technical constraints also affect real-world applicability. Sport science relies heavily on multimodal data—movement video, biomechanics, wearable-sensor signals, and health records—yet general-purpose GAI models are rarely trained on such specialised datasets, limiting their ability to capture individual differences and real-time conditions. Although some studies have attempted to integrate wearable data or computer vision modules to improve feedback precision [84], these solutions increase deployment cost and technical complexity. Furthermore, few studies provide explicit safeguards regarding data protection, privacy management, or model transparency, contributing to hesitancy among practitioners.

This review also identified several areas where the existing literature remains notably underdeveloped. First, most studies focus on technical feasibility or short-term effects, with limited investigation into the mechanisms through which GAI exerts influence in sport and exercise contexts. Second, evidence is lacking on how GAI should be adapted to different populations (e.g., youth athletes vs. older adults with chronic conditions) and sport settings (e.g., high-performance competition vs. recreational fitness). Third, the division of labour and optimal collaboration between GAI and human experts remains poorly understood. Questions such as how coaches and AI should co-design training plans, or how physical therapists should supervise and adjust AI-generated rehabilitation programs, have not yet been empirically examined. Addressing these gaps is essential for the long-term, responsible adoption of GAI in practice, yet current scholarship offers only limited insights.

### Ethical Challenges and Future Directions for the Application of GAI in Sport Science (RQ3)

The large-scale adoption of GAI in sport and exercise science raises substantial ethical and governance challenges that must accompany future technological development. Although most included studies focused on application outcomes, ethical considerations were rarely addressed explicitly. Drawing on existing ethical frameworks in sport science and AI research [96], and extending them in light of the reviewed evidence, this section focuses on ethical risks most directly affecting user safety, equity, and governance, including privacy and data protection, algorithmic bias, informed consent and autonomy, accountability, and long-term data governance.

Privacy and data governance constitute foundational concerns. Sport science applications frequently rely on sensitive multimodal data, including training load, biomechanical signals, physiological monitoring, and health records [97]. Across the reviewed studies—particularly system development and applied training or rehabilitation research—such data were commonly used for content generation, feedback, or model optimisation. However, detailed reporting on data collection, storage, secondary use, and integration into model pipelines was largely absent, raising risks related to privacy, confidentiality, and data misuse [98]. Existing legal frameworks, including health-privacy regulations and the GDPR, provide only partial protection and do not fully address the specific demands of sportrelated AI systems [99], underscoring the need for more explicit protocols.

Algorithmic bias represents a further ethical challenge. Unequal representation in training data and knowledge sources may lead to systematically different accuracy and safety profiles across populations, including women, youth athletes, older adults, para-athletes, and individuals with chronic conditions [100]. Such bias is particularly concerning in inclusive physical education and public-health contexts, where inaccurate or biased recommendations may exacerbate inequities or increase injury risk. Addressing this issue requires diversified training data and continuous bias-monitoring during deployment.

Issues of informed consent and user autonomy are also insufficiently addressed. Many studies did not clarify whether users understood the role, capabilities, and limitations of GAI in teaching, training, or rehabilitation contexts. In health-related applications, overestimation of AI decision-making capacity may lead to inappropriate reliance. User rights—including opting out of data collection, declining AI recommendations, or choosing human alternatives—are rarely articulated, indicating gaps in procedural ethics.

Accountability remains poorly defined. When AI-generated training or rehabilitation recommendations contribute to adverse outcomes, responsibility attribution among system developers, deploying institutions, practitioners, and users is unclear, potentially hindering adoption in professional settings [101, 102]. Long-term data governance raises additional concerns, as athlete performance and biometric data are often controlled by institutions or commercial entities rather than by athletes themselves, creating unresolved questions about data ownership, access rights, and future use [96].

Importantly, this review does not advocate a binary position on whether conversational GAI tools (e.g. ChatGPT) should or should not be used in sport and exercise science. Rather, the evidence suggests that GAI should be conceptualised as a decision-support technology rather than a replacement for professional expertise. Ethical use is defensible only when embedded within transparent data-governance structures, awareness of population-specific bias, informed user consent, and clearly defined human oversight and accountability mechanisms.

From a practical perspective, current evidence supports cautious and supervised integration of GAI into sport, education, and rehabilitation practice. AI-generated outputs should be implemented under expert supervision, with systematic validation required for high-risk or clinical populations. Local pilot testing in real-world settings is recommended prior to large-scale deployment, and all AI-generated recommendations should remain subject to mandatory human review.

Based on the findings of this review, the responsible development of GAI in sport and exercise science requires coordinated progress across three core domains: technology, governance, and practice.

1. Strengthening high-quality empirical research. Large-sample, multi-centre randomised controlled trials are needed to evaluate the long-term effectiveness, safety, and underlying mechanisms of GAI applications in training, rehabilitation, and health promotion, thereby providing a robust empirical basis for ethical and regulatory guidance [103].
2. Enhancing professional training and user education. As coaches, teachers, and rehabilitation practitioners typically mediate interactions between AI systems and end users, they must be equipped to interpret AI outputs, recognise risks related to bias, privacy, and over-reliance, and implement appropriate human oversight to support safe and effective human–AI collaboration [104].
3. Advancing multimodal and domain-specific GAI models. Future systems should integrate image, video, sensor, and physiological data to improve movement recognition, individualised monitoring, and adaptive feedback, thereby reducing hallucinations and enhancing domain alignment [103, 105].
4. Establishing expert-governed human–AI collaboration frameworks. Clear delineation of AI roles in content generation, risk detection, and personalisation is essential, with human experts retaining responsibility for supervision and final decision-making within an AI-assisted, expert-governed model of practice [106].
5. Developing comprehensive ethical and regulatory frameworks. Robust governance structures—including data privacy standards, model transparency, bias monitoring, human-oversight mechanisms, and accountability allocation—are required to ensure the safe, equitable, and trustworthy adoption of GAI in sport science [105].

### Limitations

Several limitations should be acknowledged. First, substantial multidimensional heterogeneity across studies precluded formal metaanalysis. In particular, pronounced outcome heterogeneity was evident, with studies assessing non-commensurable domains including physical performance (e.g., hypertrophy, maximal strength, endurance), behavioural and psychological outcomes (e.g., engagement, adherence), educational outcomes (e.g., learning efficiency), and health-related indicators (e.g., BMI and blood pressure). Moreover, incompatible outcome metrics and reporting formats—such as absolute values, percentage changes, questionnaire scores, categorical ratings, and qualitative assessments, often without sufficient statistical detail—prevented transformation into a common effect size.

Second, the synthesis of heterogeneous outcome measures represents an additional limitation, particularly in studies evaluating GAI in training and intervention contexts. Many studies assessed multiple, distinct adaptations (e.g., hypertrophy, maximal strength, endurance, and health-related indicators) and reported inconsistent effects across outcomes. Consequently, the conclusions of this review primarily reflect higher-level evidence patterns rather than adaptation-specific effects. While appropriate for a broad evidence synthesis, this approach may obscure outcome-level variability. Future reviews may therefore adopt outcome- or modality-specific syntheses to better capture differential adaptations.

Third, despite a comprehensive search strategy, the rapid evolution of GAI research means that recently published studies or preprints may not have been captured, potentially affecting the completeness of the evidence base.

Fourth, the classification of studies into application subdomains and analytical pathways involved a degree of interpretive judgment. Although independent coding and consensus procedures were used to minimise subjectivity, some interpretive bias cannot be fully excluded.

Finally, this review was restricted to peer-reviewed literature. The exclusion of grey literature and industry reports may have led to an underrepresentation of emerging applied systems or unpublished negative findings.

## CONCLUSIONS

This review synthesised current evidence on the application of GAI in sport and exercise science. Although GAI demonstrates considerable promise—supporting the generation of training and rehabilitation plans aligned with established principles, providing interdisciplinary knowledge integration, and enhancing physical education, coaching, and health-management practices—as of the time covered by this review, it has not yet reached a stage of mature or reliable deployment. Limitations in study quality and consistency, together with persistent shortcomings in individualisation and safety assurance within GAI-generated content, indicate that findings should be interpreted with caution. Overall, this review provides a systematic evaluation of GAI applications in sport science and outlines key directions for advancing the field.

Future research should prioritise several areas.

First, large-scale, multi-centre empirical studies—including randomised controlled trials and long-term follow-up—are needed to establish the efficacy, safety, and mechanisms of GAI-supported interventions in training, rehabilitation, and health promotion.

Second, professional training and ethical governance should be strengthened. Improving AI literacy among teachers, coaches, and clinical practitioners, alongside clear standards for data privacy and responsibility allocation, will be essential for building trust and ensuring safe implementation.

Third, advances in multimodal technologies and model optimisation are required. Integrating movement imagery, sensor-derived biomechanical data, and physiological signals into GAI systems will enhance the precision of personalised recommendations and real-time feedback.

## Supplementary Material

Is generative artificial intelligence catalyzing a paradigmatic transformation in sport science? - Evidence from a mixed-methods systematic review

## Data Availability

All the data used in this study came from publicly available databases.

## Availability of Data and Material

All data generated or analysed during this study are included in this published article and its supplementary information files (LINK).

## Registration

Registered on PROSPERO (registration number: CRD420251184213).

## List of Abbreviations

GAI Generative artificial intelligence, LDA Latent Dirichlet Allocation.
