# Supplementary material for: Is generative artificial intelligence catalyzing a paradigmatic transformation in sport science? – Evidence from a mixed-methods systematic review
Source: Biol Sport. 2026 Aug 24;43:1693–712. doi: 10.5114/biolsport.2026.162050 (PMC13712261; doi:10.5114/biolsport.2026.162050)
Supplement: Supplementary file 1 [file JBS-43-58110-s1.pdf]

Supplementary Material
to paper DOI: https://doi.org/10.5114/biolsport.2026.162050

Is Generative Artificial Intelligence Catalyzing a Paradigmatic Transformation in Sport Science?— Evidence from a Mixed-Methods Systematic Review

Catalogue

Appendix A PRISMA Checklist 1

Appendix B Topic search queries used for data collection 4

Appendix C Included in the literature characteristics table 9

Appendix D Methodological Quality Assessment 24

RCT (n = 2) 25

Quasi-experimental (n = 5) 26

Cross-sectional (n = 31) 29

Qualitative study (n = 7) 44

Summary 48

Appendix E LDA Modeling Code 50

Supplementary Material
to paper DOI: https://doi.org/10.5114/biolsport.2026.162050

Appendix A PRISMA Checklist

Due to typesetting issues, the page will be forwarded to the next page.

Section and Topic	Item #	Checklist item	Location where item is reported
**TITLE**			
Title	1	Identify the report as a systematic review.	1
**ABSTRACT**			
Abstract	2	See the PRISMA 2020 for Abstracts checklist.	1
**INTRODUCTION**			
Rationale	3	Describe the rationale for the review in the context of existing knowledge.	1-3
Objectives	4	Provide an explicit statement of the objective(s) or question(s) the review addresses.	1-3
**METHODS**			
Eligibility criteria	5	Specify the inclusion and exclusion criteria for the review and how studies were grouped for the syntheses.	4
Information sources	6	Specify all databases, registers, websites, organisations, reference lists and other sources searched or consulted to identify studies. Specify the date when each source was last searched or consulted.	5
Search strategy	7	Present the full search strategies for all databases, registers and websites, including any filters and limits used.	5
Selection process	8	Specify the methods used to decide whether a study met the inclusion criteria of the review, including how many reviewers screened each record and each report retrieved, whether they worked independently, and if applicable, details of automation tools used in the process.	5
Data collection process	9	Specify the methods used to collect data from reports, including how many reviewers collected data from each report, whether they worked independently, any processes for obtaining or confirming data from study investigators, and if applicable, details of automation tools used in the process.	6
Data items	10a	List and define all outcomes for which data were sought. Specify whether all results that were compatible with each outcome domain in each study were sought (e.g. for all measures, time points, analyses), and if not, the methods used to decide which results to collect.	6
	10b	List and define all other variables for which data were sought (e.g. participant and intervention characteristics, funding sources). Describe any assumptions made about any missing or unclear information.	6

Study risk of bias assessment	11	Specify the methods used to assess risk of bias in the included studies, including details of the tool(s) used, how many reviewers assessed each study and whether they worked independently, and if applicable, details of automation tools used in the process.	6
Effect measures	12	Specify for each outcome the effect measure(s) (e.g. risk ratio, mean difference) used in the synthesis or presentation of results.	6
Synthesis methods	13a	Describe the processes used to decide which studies were eligible for each synthesis (e.g. tabulating the study intervention characteristics and comparing against the planned groups for each synthesis (item #5)).	6-8
	13b	Describe any methods required to prepare the data for presentation or synthesis, such as handling of missing summary statistics, or data conversions.	6-8
	13c	Describe any methods used to tabulate or visually display results of individual studies and syntheses.	6-8
	13d	Describe any methods used to synthesize results and provide a rationale for the choice(s). If meta-analysis was performed, describe the model(s), method(s) to identify the presence and extent of statistical heterogeneity, and software package(s) used.	6-8
	13e	Describe any methods used to explore possible causes of heterogeneity among study results (e.g. subgroup analysis, meta-regression).	6-8
	13f	Describe any sensitivity analyses conducted to assess robustness of the synthesized results.	6-8
Reporting bias assessment	14	Describe any methods used to assess risk of bias due to missing results in a synthesis (arising from reporting biases).	6
Certainty assessment	15	Describe any methods used to assess certainty (or confidence) in the body of evidence for an outcome.	6-8
**RESULTS**			
Study selection	16a	Describe the results of the search and selection process, from the number of records identified in the search to the number of studies included in the review, ideally using a flow diagram.	8
	16b	Cite studies that might appear to meet the inclusion criteria, but which were excluded, and explain why they were excluded.	8
Study characteristics	17	Cite each included study and present its characteristics.	9
Risk of bias in studies	18	Present assessments of risk of bias for each included study.	10
Results of individual studies	19	For all outcomes, present, for each study: (a) summary statistics for each group (where appropriate) and (b) an effect estimate and its precision (e.g. confidence/credible interval), ideally using structured tables or plots.	10
Results of	20a	For each synthesis, briefly summarise the characteristics and risk of bias among contributing studies.	12-20

syntheses	20b	Present results of all statistical syntheses conducted. If meta-analysis was done, present for each the summary estimate and its precision (e.g. confidence/credible interval) and measures of statistical heterogeneity. If comparing groups, describe the direction of the effect.	12-20
	20c	Present results of all investigations of possible causes of heterogeneity among study results.	12-20
	20d	Present results of all sensitivity analyses conducted to assess the robustness of the synthesized results.	12-20
Reporting biases	21	Present assessments of risk of bias due to missing results (arising from reporting biases) for each synthesis assessed.	10
Certainty of evidence	22	Present assessments of certainty (or confidence) in the body of evidence for each outcome assessed.	16-20
**DISCUSSION**			
Discussion	23a	Provide a general interpretation of the results in the context of other evidence.	20-25
	23b	Discuss any limitations of the evidence included in the review.	20-25
	23c	Discuss any limitations of the review processes used.	20-25
	23d	Discuss implications of the results for practice, policy, and future research.	20-25
**OTHER INFORMATION**			
Registration and protocol	24a	Provide registration information for the review, including register name and registration number, or state that the review was not registered.	4
	24b	Indicate where the review protocol can be accessed, or state that a protocol was not prepared.	4
	24c	Describe and explain any amendments to information provided at registration or in the protocol.	4
Support	25	Describe sources of financial or non-financial support for the review, and the role of the funders or sponsors in the review.	27
Competing interests	26	Declare any competing interests of review authors.	27
Availability of data, code and other materials	27	Report which of the following are publicly available and where they can be found: template data collection forms; data extracted from included studies; data used for all analyses; analytic code; any other materials used in the review.	27

From: Page MJ, McKenzie JE, Bossuyt PM, Boutron I, Hoffmann TC, Mulrow CD, et al. The PRISMA 2020 statement: an updated guideline for reporting systematic reviews. BMJ 2021;372:n71. doi: 10.1136/bmj.n71. This work is licensed under CC BY 4.0. To view acopy of this license, visit

Appendix B Topic search queries used for data collection

**Web of Science Core Collection® data base**		
# 1	748496	("sport science" OR "exercise science" OR "kinesiology" OR "physical education" OR "PE" OR "sport training" OR "athletic training" OR "sport performance" OR "rehabilitation" OR "exercise therapy" OR "health promotion" OR "fitness" OR "biomechanics" OR "motor learning" OR "motor control" OR "sport pedagogy" OR "movement analysis") (Topic)<br>Index =SCI-EXPANDED (1995-present), SSCI (1990-present), AHCI (2004-2011), CPCI-S (1991-present), CPCI-SSH (1991-present), CCR-EXPANDED (1985-present), and IC (1993-present)
# 2	80962	("generative artificial intelligence" OR "generative AI" OR "large language model*" OR "LLM*" OR "ChatGPT" OR "GPT-4" OR "Gemini" OR "Bard" OR "Claude" OR "ERNIE Bot" OR "Sora" OR "Kimi" OR "DeepSeek" OR "Grok" OR "AI-generated content" OR "AIGC" OR "GPT-5") (Topic)<br>Index =SCI-EXPANDED (1995-present), SSCI (1990-present), AHCI (2004-2011), CPCI-S (1991-present), CPCI-SSH (1991-present), CCR-EXPANDED (1985-present), and IC (1993-present)
#3	480	#1 AND #2<br>Index =SCI-EXPANDED (1995-present), SSCI (1990-present), AHCI (2004-2011), CPCI-S (1991-present), CPCI-SSH (1991-present), CCR-EXPANDED (1985-present), and IC (1993-present)

#4	432	#3 and Article or Proceeding Paper or Early Access (Document Types) Index =SCI-EXPANDED (1995-present), SSCI (1990-present), AHCI (2004-2011), CPCI-S (1991-present), CPCI-SSH (1991-present), CCR-EXPANDED (1985-present), and IC (1993-present)

PubMed (including MEDLINE and PubMed Central)		
# 1	177	((((((((((((((generative artificial intelligence[MeSH Major Topic]) OR (generative AI[MeSH Major Topic])) OR (large language model*[MeSH Major Topic])) OR (LLM[MeSH Major Topic])) OR (ChatGPT[MeSH Major Topic])) OR (GPT-4[MeSHMajor Topic])) OR (Gemini[MeSH Major Topic])) OR (Bard[MeSH Major Topic])) OR (Claude[MeSH Major Topic])) OR (ERNIE Bot[MeSH Major Topic])) OR (Sora[MeSH Major Topic])) OR (Kimi[MeSH Major Topic])) OR (DeepSeek[MeSH Major Topic])) OR (Grok[MeSH Major Topic])) OR (Grok[MeSH Major Topic])) OR (AI-generated content[MeSH Major Topic])) OR (AIGC[MeSH Major Topic]) OR (GPT-5[MeSH Major Topic])
# 2	406657	(((((((((((((((sport science[MeSH Major Topic]) OR (exercise science[MeSH Major Topic])) OR (kinesiology[MeSH Major Topic])) OR (physical education[MeSH Major Topic])) OR (PE[MeSH Major Topic])) OR (sport training[MeSH Major Topic])) OR(athletic training[MeSH Major Topic])) OR (sport performance[MeSH Major Topic])) OR (rehabilitation[MeSH Major Topic])) OR (exercise therapy[MeSH Major Topic])) OR (health promotion[MeSH Major Topic])) OR (fitness[MeSH Major Topic])) OR (biomechanics[MeSH Major Topic])) OR (motor learning[MeSH Major Topic])) OR (motor control[MeSH Major Topic])) OR (sport pedagogy[MeSH Major Topic])) OR (movement analysis[MeSH Major Topic])
#3	3	#1 AND #2

SPORTDiscus		
# 1	18	SU ("generative artificial intelligence" OR "generative AI" OR "large language model*" OR "LLM*" OR "ChatGPT" OR "GPT-4" OR "Gemini" OR "Bard" OR "Claude" OR "ERNIE Bot" OR "Sora" OR "Kimi" OR "DeepSeek" OR "Grok" OR "AI-generated content" OR "AIGC" OR "GPT-5") AND SU ("sport science" OR "exercise science" OR "kinesiology" OR "physical education" OR "PE" OR "sport training" OR "athletic training" OR "sport performance" OR "rehabilitation" OR "exercise therapy" OR "health promotion" OR "fitness" OR "biomechanics" OR "motor learning" OR "motor control" OR "sport pedagogy" OR "movement analysis")

**Scopus**		
# 1	1263	TITLE-ABS-KEY ("sport science" OR "exercise science" OR "kinesiology" OR "physical education" OR "PE" OR "sport training" OR "athletic training" OR "sport performance" OR "rehabilitation" OR "exercise therapy" OR "health promotion" OR "fitness" OR "biomechanics" OR "motor learning" OR "motor control" OR "sport pedagogy" OR "movement analysis") AND TITLE-ABS-KEY ("generative artificial intelligence" OR "generative AI" OR "large language model*" OR "LLM*" OR "ChatGPT" OR "GPT-4" OR "Gemini" OR "Bard" OR "Claude" OR "ERNIE Bot" OR "Sora" OR "Kimi" OR "DeepSeek" OR "Grok" OR "AI-generated content" OR "AIGC" OR "GPT-5")
# 2	1063	#1 AND (LIMIT-TO (DOCTYPE , "ar") OR LIMIT-TO (DOCTYPE , "cp") OR LIMIT-TO (DOCTYPE , "cr"))

Appendix C Included in the literature characteristics table

Author	Study design	GAI models	Prompt structure (Information granularity)	Evaluation source	Evaluation focus	Intervention or application method	Key findings	Bias risk assessment
Genç N (2023) [34]	Qualitative study	ChatGPT -3.5	Semi-structured (Low to Moderate)	Researchers	Potential Applications; Benefits; and Risks	A semi-structured qualitative design was used in which the researcher posed predefined questions to ChatGPT and analysed its responses via content analysis to explore applications in physical education and sport.	ChatGPT demonstrated potential utility in personalised training, performance analysis, lesson planning, and educational support, while also presenting risks related to information accuracy, over-reliance on technology, and ethical concerns.	Moderate
Miao C (2025) [40]	RCT	ChatGPT -4	Structured (High)	Students	Effectiveness ; Usability; Engagement	A crossover experimental design compared AI-assisted football tactical instruction integrating ChatGPT-based language guidance with Metrica PlayBase visual analysis against traditional coach-led instruction among male college students.	AI-assisted instruction produced significantly greater improvements in tactical knowledge comprehension, tactical decision-making ability, and learning satisfaction than traditional instruction ($p<.001$), with large effect sizes and no carryover effects.	Low
Dekerlegand R (2025) [36]	Cross-sectional study	Unspecified model	Not Applicable	Students	Usability; Acceptance; Accuracy; Safety	A mixed-methods, cross-sectional survey based on the Technology Acceptance Model and UTAUT was used to examine rehabilitation sciences students' acceptance, usage patterns, and perceptions of generative AI tools (primarily ChatGPT) in education.	Over half (56.1%) of students reported using generative AI for learning-related tasks, with users perceiving significantly greater usefulness and ease of use than non-users, while concerns about accuracy, overreliance, and impact on deeper learning remained prevalent.	Low
Prosen M (2025) [37]	Cross-sectional study	Unspecified model	Not Applicable	Students	Usability; Perceived usefulness; Ethics; Privacy	A descriptive cross-sectional survey using a validated 27-item SAAIHE scale examined health sciences students' usage patterns, perceptions, ethical concerns, and integration of generative AI in academic learning.	Students showed generally positive attitudes toward generative AI, with perceived usefulness strongly predicted by frequency, duration, skill, and confidence of use, alongside persistent concerns about ethics, privacy, and equitable integration.	Low
Wu Q (2025) [38]	Cross-sectional study	Unspecified model	Not Applicable	Students	Usability; Acceptance; Behavioural intention; Perceived risk	Application of AIGC in university physical education, examined through an extended UTAUT model using a large-scale questionnaire survey and PLS-SEM analysis.	Performance expectancy, social influence, facilitating conditions, and perceived risk significantly predicted behavioural intention, which in turn strongly predicted actual AIGC use, while effort expectancy and gender moderation effects were non-significant.	Low

Sapre T (2025) [42]	Qualitative study	ChatGPT -3.5	Structured, Fixed Q&A Prompts	Experts	Accuracy; Usability; Personalisation; Safety	ChatGPT-3.5 was used within a rapid-cycle adaptation framework to generate and refine concise, step-by-step exercise instructions and cue words for the InPACT at Home program, guided by Universal Design for Learning principles and expert review.	AI-assisted adaptation enabled the scalable development of accessible instructional resources for neurodivergent children, improving instructional clarity and inclusivity, although effectiveness on physical activity outcomes has yet to be empirically tested.	Low
Qiu Y (2024) [41]	Quasi-experimental study	ChatGPT; ERNIE Bot	Semi-structured (Moderate)	Performance tests & Expert coaches	Accuracy; Safety; Effectiveness; Consistency	A 16-week quasi-experimental study comparing self-guided badminton skill learning using LLM-based applications (ChatGPT/New Bing) with traditional teacher-led instruction among novice university students.	Although both groups improved significantly, the LLM-based group showed inferior performance in complex skills (clear and smash), with qualitative and quantitative evidence indicating that LLM hallucinations generated inaccurate technical feedback that negatively affected motor skill acquisition.	Low
Chang S H (2025) [43]	Cross-sectional study	ChatGPT	Not Applicable	Students	Usability; Trust; Perceived risks; Inclusion (DEI)	Cross-sectional survey applying an extended Technology Acceptance Model to examine university students' perceptions of using ChatGPT to support inclusion in sport education, with regression analyses predicting perceived opportunities and challenges.	Trust and frequency of ChatGPT use significantly predicted perceived opportunities for inclusion, whereas concerns and gender predicted perceived challenges, indicating a dual perception in which inclusive potential coexists with ethical and representational risks.	Low
Ma W (2025) [35]	System development and validation study	Custom system based on ChatGPT -4	Structured (High)	Objective metrics & Expert coaches	Accuracy; Effectiveness; Consistency; Personalization	Development and evaluation of an AI table tennis coaching system integrating computer vision, motion capture (OpenPose), and a multimodal large language model (GPT-4) with a domain-specific table tennis knowledge base to detect errors and provide personalized training guidance for beginners.	The multimodal LLM-based coaching system achieved substantially higher error-recognition accuracy than direct GPT-4 use (average accuracy 0.67 vs. 0.43), with particularly strong performance for arm- and racket-related errors, and was rated by experts as accurate, comprehensible, and cost-effective for novice table tennis training.	Low

Albaloul O (2026) [33]	Cross-sectional study	ChatGPT-4.5; ChatGPT-4o; ChatGPT-o3; ChatGPT-o4-mini; ChatGPT-o4-mini-high	Unstructured (Moderate)	Objective metrics & Expert coaches	Accuracy	Five ChatGPT versions answered a validated 36-item basketball CCK test and generated explanations, which were evaluated by experts.	All ChatGPT versions achieved high accuracy (80.6–94.4%) with no significant differences across models (p = .107), and 100% of explanations were judged appropriate by experts.	Low
Kohiyama K (2025) [39]	Quasi-experimental study	Custom system based on ChatGPT-4	Semi-structured (Moderate)	Students	Learning effectiveness; Learning behaviors and engagement patterns	OT students used a ChatGPT-based bot for one month to generate and practice MCQs in anatomy, kinesiology, and physiology during self-study.	Mock exam scores improved significantly after one month (mean gain +5.0, d = 0.83), and cluster analysis showed that greater total usage time—not question quantity—was associated with the largest performance gains.	Moderate
Chung S M (2024) [69]	Cross-sectional study	ChatGPT-4	Fixed Q&A prompts (Low)	Clinicians	Accuracy; Safety; Utility	Expert evaluation of ChatGPT-4 responses to 14 frequently asked exercise-related questions for patients with type 2 diabetes, assessed using Likert scales for validity, safety, and utility against ADA and ACSM guidelines.	ChatGPT provided largely accurate, safe, and useful exercise information, but 28.6% of responses were incomplete, indicating it is suitable as a supplementary educational tool rather than a replacement for professional guidance.	Low
Xu Y (2024) [71]	Cross-sectional study	ChatGPT-4	Structured (High)	Experts	Accuracy; Comprehensiveness; Applicability	Comparative expert evaluation of personalized exercise prescriptions generated by ChatGPT versus an Intelligent Health Promotion System (IHPS), using real patient data structured by the Transtheoretical Model and rated on accuracy, comprehensiveness, and applicability.	ChatGPT generally outperformed IHPS in accuracy, comprehensiveness, and applicability, but posed higher health risks in patients with functional movement disorders.	Low

Larbi D (2025) [81]	System development and validation study	ChatGPT-3.5	Semi-structured (Moderate)	Users	Usability; User experience	Design and usability testing of "FysBot," a physical activity app integrating a ChatGPT-based chatbot with persona-driven prompting, step-count integration, and exercise recommendations, evaluated using think-aloud sessions.	Participants valued the chatbot's motivational exercise recommendations and instructions, but usability issues, limited personalisation, and occasional inaccuracies (e.g., step counts and follow-up responses) constrained effectiveness.	Low
Philuek P (2025) [75]	RCT	ChatGPT-4	Structured (Low)	Performance tests	Effectiveness	An 8-week randomized controlled pilot intervention in which ChatGPT-4.0 generated a structured aerobic, resistance, and flexibility exercise program performed three times per week by healthy overweight young adults.	Compared with controls, the ChatGPT-generated exercise program significantly reduced BMI and post-exercise heart rate and improved muscular endurance, while pre–post analyses showed additional benefits in body composition and cardiovascular fitness.	Moderate
Chittimalla S K (2025) [87]	System development and validation study	ChatGPT-4	Not applicable	Objective metrics	Accuracy; Robustness	Integration of a CNN-based yoga pose recognition model with a LLM and real-world datasets (e.g., Yoga-82) to provide contextualized pose interpretation and personalized feedback for fitness assistance.	The multimodal LLM-enhanced system achieved higher pose recognition accuracy (up to 92%) and greater robustness across environmental conditions, while improving personalized feedback and user satisfaction compared with conventional vision-only approaches.	Low
Saraç H (2025) [73]	Cross-sectional study	ChatGPT-4; ChatGPT-4o; Gemini-1.5 Pro	Structured, FITT (High)	Expert coaches	Accuracy; Safety	Comparison of personalized gym-based exercise programs generated by three GAI chatbots versus human expert trainers for a hypothetical obese individual, evaluated against ACSM and NASM guidelines using a standardized FITT-based prompt.	AI chatbot–generated programs were largely guideline-consistent and rapidly produced, but showed notable discrepancies in initial load and target heart rate prescriptions compared with expert trainers.	Low
Khasentino J (2025) [84]	System development and validation study	Custom system based on Gemini Ultra 1.0	Structured (High)	Objective metrics & Experts	Accuracy; Personalisation; Safety	A Gemini-based LLM (PH-LLM) fine-tuned to integrate aggregated wearable sensor data with textual reasoning to provide personalized sleep and fitness coaching, expert-level knowledge assessment, and patient-reported outcome prediction.	PH-LLM matched or exceeded human expert performance in sleep and fitness examinations, produced high-quality personalized coaching comparable to experts, and accurately predicted self-reported sleep outcomes from wearable data.	Low

Priyanka S P (2025) [86]	System development and validation study	Gemini LLM	Unstructured (Low)	Objective metrics	Accuracy; Effectiveness	A computer-vision–based virtual fitness trainer integrating OpenCV and MediaPipe for real-time pose estimation with a Gemini LLM to generate personalized, context-aware exercise feedback during home workouts.	The system achieved over 95% accuracy in landmark detection, improved exercise form and user engagement with real-time feedback.	Low
Bak M (2024) [90]	Cross-sectional study	ChatGPT-3.5; Google Bard; Llama 2	Fixed Q&A prompts (Low)	Experts	Accuracy; Personalisation	Scenario-based evaluation of three LLM-powered generative conversational agents using validated Transtheoretical Model scenarios to assess their ability to identify users' motivation states and provide stage-appropriate behavior change guidance.	The model can identify and support individuals in the preparation, action, and maintenance phases, but its ability to recognize those in the unintentional or hesitant stages is weak, covering only 20–30% of the motivational process.	Low
Dergaa I (2024) [77]	Cross-sectional study	ChatGPT-4	Structured, FITT (High)	Experts	Accuracy; Safety; Effectiveness; Personalisation	A simulation study in which GPT-4 generated individualized exercise prescriptions for multiple hypothetical individuals with diverse health conditions, structured according to FITT principles and evaluated by expert exercise scientists.	GPT-4 demonstrated a solid grasp of general exercise prescription principles and produced broadly appropriate programs, but showed limitations in individualized intensity prescription, progression, safety considerations, and contextual factors.	Low
Kothari A (2025) [74]	System development and validation study	Custom system based on Llama 3 8B	Semi-structured (High)	Objective metrics & Users	Usability; Personalisation	An AI-driven wellness platform using a LoRA-fine-tuned and quantized Llama 3 8B LLM that integrates user-provided prompts with real-time Apple HealthKit data to generate personalized diet and fitness recommendations on edge devices.	The system produced context-aware, guideline-consistent diet and exercise recommendations aligned with individual preferences and activity levels.	Low

Arumilli A (2024) [89]	Cross-sectional study	ChatGPT-3.5; Llama-2 7B; FLAN-T5; Gemini 1.5 Pro; Gemma 7B; Mistral 7B	Fixed Q&A prompts (Low)	Objective metrics	Accuracy	Few-shot application of multiple LLMs to extract and classify exercise type, intensity, duration, and post-exercise feelings from free-text exercise logs, compared against traditional transformer classifiers.	LLMs substantially outperformed BERT-, RoBERTa-, and XLNet-based models in multi-dimensional exercise log classification, with GPT-3.5 achieving the highest overall accuracy.	Low
Fu T H (2023) [91]	System development and validation study	ChatGPT-3; Stable Diffusion	Unstructured (Low)	Objective metrics	Effectiveness; Usability	An AI-assisted fitness exergame incorporating a generative virtual coach and an empowering game mechanism was applied to guide repetitive strength exercises and enhance player engagement through dynamic, co-created game content.	A single-group pre–post experiment showed significant improvements in intrinsic motivation and engagement (interest/enjoyment, value/usefulness, perceived choice, and overall motivation) ($p<0.05$).	Low
Zaleski A L (2024) [70]	Cross-sectional study	ChatGPT-3.5	Unstructured (Low)	Experts	Accuracy; Safety; Usability	ChatGPT was prompted with standardized open-ended queries to generate individualized exercise recommendations for 26 clinical populations, which were evaluated against ACSM guidelines.	AI-generated exercise recommendations showed high accuracy (90.7%) but low comprehensiveness (41.2%) and college-level readability, with notable gaps in FITT components and overemphasis on medical clearance.	Low
Willms A (2024) [83]	Qualitative study	ChatGPT-3	Structured (Low)	Researchers	Usability; Accuracy	ChatGPT-3 was used with theory-informed, structured prompts grounded in the Multi-Process Action Control framework to rapidly generate tailored content for a family-based physical activity just-in-time adaptive intervention delivered via a no-code mHealth platform.	ChatGPT enabled efficient generation of 13 theory-aligned intervention lessons within two months and was deemed acceptable and easy to use, but required expert review due to issues with reproducibility, occasional inaccuracies, and fabricated references.	Low
Bhattacharya H (2024) [88]	System development and validation study	Custom system based on Google PaLM 2	Semi-structured (High)	Objective metrics	Accuracy	Design and implementation of a GPT-powered AI gym trainer and fitness tracker that generates personalized workout and nutrition plans using prompt engineering techniques integrated into a full-stack mobile application.	The GPT-based system successfully produced adaptive, personalized fitness and diet plans, with few-shot prompting substantially improving output quality and consistency.	Low

Kamnerddee C (2024) [85]	System development and validation study	Custom system based on LLM et al.	Structured	Objective metrics & Users	Usability; User experience	Comparative A/B usability testing of a fitness app UI prototype designed by human UX/UI designers versus a generative-AI tool (Visily.ai) integrated into the Design Thinking process, assessed using SUS and qualitative interviews.	Human-created and AI-generated prototypes showed no statistically significant difference in SUS scores, but users consistently preferred the human-designed interface for intuitiveness and engagement, while AI-generated designs were perceived as efficient and simple yet less context-aware and less engaging over time.	Low
Akrimi S (2025) [78]	Cross-sectional study	ChatGPT-4o	Structured, FITT (High)	Expert coaches	Safety; Effectiveness	Generation of 12-week exercise plans for fictional patients with type 2 diabetes using ChatGPT under three prompt conditions of increasing detail, followed by expert evaluation of safety and training quality based on ADA and ACSM guidelines.	ChatGPT-generated exercise plans frequently exhibited safety and feasibility shortcomings—especially for patients with secondary complications—although more detailed prompts modestly improved quality.	Low
Li G (2025) [72]	Cross-sectional study	ChatGPT-4	Structured, FITT (High)	Experts	Personalization; Effectiveness; Safety; Comprehensiveness	Comparison of a 16-week personalized weight-loss and fitness program generated by GPT-4 versus plans designed by three certified human coaches, evaluated by blinded experts using FITT-based criteria.	GPT-4 demonstrated comparable overall performance to human coaches, with higher personalization scores but no statistically significant differences in effectiveness, safety, or comprehensiveness.	Low
Vitale J (2025) [79]	Cross-sectional study	ChatGPT-3.5; ChatGPT-4; Gemini Bard	Unstructured (Low)	Experts	Validity; Reliability	ChatGPT-3.5, ChatGPT-4, and Google Bard were prompted with standardized expert-style instructions to answer ten predefined FAQs on athlete sleep and jet lag, with responses independently evaluated by a multidisciplinary expert panel using content validity and reliability metrics.	ChatGPT-4 demonstrated the highest content validity and overall expert preference, performing well for sleep advice but showing that all LLMs—especially Google Bard—had limited reliability and accuracy for complex jet lag recommendations, necessitating expert oversight.	Low

Solomon T P J（2025）[80]	Cross-sectional study	ChatGPT-4o; ChatGPT-4o mini; Gemini 1.5 Flash; Gemini 1.5 Pro; Claude 3.5 Sonnet; Claude 3 Haiku	Unstructured (Low) vs. Structured (High)	Experts	Accuracy; Completeness; Clarity; Evidence quality; Consistency	Publicly available LLM-based chatbots were evaluated using simple vs. detailed zero-shot prompts and a sports nutrition MCQ exam to assess accuracy, completeness, clarity, evidence quality, and test–retest reliability.	LLM chatbots showed acceptable test–retest reliability but only moderate and model-dependent accuracy, with better performance for carbohydrate-related guidance than for nuanced topics (e.g., energy availability, hydration, supplements).	Low
Onan D (2025) [82]	Qualitative study	ChatGPT-4	Structured, FITT (High)	Physiotherapists & Physicians	Clinical appropriateness; Safety; Progression; Individualisation	ChatGPT-4.0 generated detailed 8-week exercise programs for lumbar disc herniation, chronic migraine, and urge urinary incontinence based on structured clinical case prompts.	ChatGPT-4.0 produced literature-aligned exercise programs but showed limitations in individualised progression and safety (e.g., overly rapid intensity increases, potentially inappropriate exercises).	Moderate
Farías H (2025) [76]	System development and validation study	Mistral-7B (SELF-RAG–based custom system); ChatGPT-4o mini	Structured (High)	Healthcare professionals	Accuracy; Relevance; Consistency; Tone	Developed a KOA adherence-support chatbot by grounding responses in guideline-based knowledge via SELF-RAG with CoT, then iteratively improved output tone through an added workflow node and re-evaluated against ChatGPT.	The post-improvement chatbot achieved higher satisfaction/median scores and reduced dispersion across clinical categories compared with the pre-improvement version and generally outperformed or matched ChatGPT, with statistically significant gains reported for key categories (e.g., NPT/PT).	Low
Villarreal-Espinosa J B (2024) [59]	Cross-sectional study	ChatGPT-4	Fixed Q&A prompts (Low)	Clinicians	Accuracy; Consistency	Ten ACL surgery-related FAQs were posed to ChatGPT, and responses were independently rated for accuracy and reproducibility by fellowship-trained orthopedic surgeons using a Likert scale.	Half of the responses were rated as completely accurate by both surgeons and 80% were reproducible over time.	Low

Bu L (2025) [63]	System development and validation study	ChatGPT-4o	Structured (High)	Objective metrics & Users	Effectiveness; Personalisation; Usability; Acceptance	An intelligent glove integrating VR, embedded sensors, and an AIGC module to generate personalized, real-time adjusted rehabilitation training scenarios based on multimodal user data.	Compared with non-AIGC control conditions, the AIGC-driven system significantly improved task progression and hand force output and showed higher user attitudes and behavioral intention.	Low
Gürses Ö A (2025) [12]	Cross-sectional study	ChatGPT-4o; Gemini Advanced	Structured (High)	Objective metrics & Physiotherapists	Accuracy; Personalisation; Consistency; Safety	ChatGPT-4o and Gemini Advanced used individualized clinical data from 40 knee osteoarthritis patients, and their generated physiotherapy programs were compared with consensus programs developed by experienced physiotherapists across 50 rehabilitation parameters.	ChatGPT-4o and Gemini Advanced achieved 74% and 70% agreement with expert consensus respectively, showing good alignment for general rehabilitation components but consistent deficiencies in exercise specificity, dosage, progression criteria, and clinical reasoning.	Low
Quinn M (2024) [60]	Cross-sectional study	ChatGPT-4; Gemini Bard	Structured (High)	Clinicians	Accuracy; Clarity; Completeness; Evidence quality; Consistency	ChatGPT-4 and Gemini were prompted with standardized questions derived from all 15 items of the 2022 AAOS clinical practice guidelines for ACL reconstruction, and responses were blindly evaluated by fellowship-trained sports medicine surgeons.	Both LLMs generated guideline-aligned and accurate responses (mean scores >4/5), but Gemini demonstrated significantly superior clarity overall and greater clarity and completeness in rehabilitation and prevention domains.	Low
McBee J C (2024) [62]	Qualitative study	ChatGPT-4	Structured (High)	Researchers	Accuracy; Comprehensiveness; Interdisciplinary reasoning; Personalisation; Safety; Ethics awareness	ChatGPT-4 was deployed via a novel multirole simulation framework to role-play a physiotherapist, psychologist, nutritionist, AI expert, athlete, and moderator in panel discussions addressing interdisciplinary questions on chatbot use in sports rehabilitation.	ChatGPT demonstrates strong interdisciplinary reasoning and educational potential, offering 24/7 support, personalized recommendations, and data tracking. However, it exhibits biases in emotion recognition, privacy risks, and imbalances in subject knowledge	Moderate

Saglam S (2025) [64]	Cross-sectional study	ChatGPT-4; ChatGPT-3.5	Structured (High)	Clinicians & Physiotherapists	Accuracy; Treatment appropriateness; Rehabilitation suitability; Consistency; Reliability	GPT-4 and GPT-3.5 generated diagnostic, treatment, surgical, and rehabilitation recommendations for 10 standardized sports-related musculoskeletal scenarios, which were blindly evaluated by 56 sports surgeons and physiotherapists using Likert-scale criteria.	GPT-4 significantly outperformed GPT-3.5 across diagnostic accuracy, treatment suitability, surgical detail, and rehabilitation appropriateness ($p<0.001$), demonstrating greater reliability and clinical relevance.	Low
Yang Q (2024) [65]	System development and validation study	Custom system based on Stable Diffusion and ControlNet	Semi-structured (High)	Students	Usability; Clarity; Effectiveness; User satisfaction	A web-based AI-assisted platform (ExPose) integrating Stable Diffusion and ControlNet to convert textual rehabilitation prescriptions, sketches, or skeletal inputs into editable exercise illustrations through canvas- and skeleton-based interaction modes.	User evaluation with 124 participants showed high usability and satisfaction (≈80% satisfied or very satisfied), but remaining limitations in customization and real-time interactivity.	Low
Vieira C (2024) [14]	Qualitative study	ChatGPT-3.5	Unstructured (Low)	Patients & Researchers	Personalisation; Usability; Engagement	ChatGPT was used to analyze qualitative patient interviews and generate patient-specific narrative concepts to support the design of personalized serious games for upper-limb motor rehabilitation.	ChatGPT can generate personalized, emotionally resonant game narratives based on patient characteristics, enhancing motivation, immersion, and rehabilitation engagement.	Moderate
Tang J (2025) [67]	System development and validation study	ChatGPT-4o	Structured (High)	Objective metrics & Experts	Accuracy; Consistency; Reliability; Interpretability; Effectiveness	Pre-trained LLMs were combined with exercise-specific features extracted from skeletal joint data and advanced prompt engineering (few-shot, chain-of-thought, certainty, role-play) to assess rehabilitation exercise quality and generate natural-language feedback.	The LLM-based approach achieved competitive accuracy for exercise quality assessment with few-shot prompting and successfully generated interpretable, clinician-like feedback, though performance remained lower than deep-learning models on some datasets and reproducibility and overconfidence issues persisted.	Low
Sun J (2024) [66]	System development and validation study	Custom system based on Qwen-14B-Chat	Structured (High)	Objective metrics & Clinicians	Accuracy; Consistency; Personalisation; Professionalism; Safety	An intelligent Rehabilitation Exercise Guidance System integrating BlazePose-based spatiotemporal action quality assessment with a LoRA-fine-tuned Qwen-14B LLM to deliver personalized, assessment-informed rehabilitation exercise consultation.	The system achieved high accuracy in temporal (deviation <10%, repetition recognition >89%) and spatial (>87%) exercise assessment and outperformed baseline LLMs (Qwen-14B-Chat and ChatGPT-4) in consultation consistency and professionalism.	Low

Safran E (2025) [61]	Cross-sectional study	ChatGPT-4	Structured (High)	Physiotherapists	Accuracy; Guideline adherence; Clarity; Completeness; Consistency	ChatGPT-4 was prompted with 20 physiotherapy-specific musculoskeletal questions and its responses were benchmarked against evidence-based clinical practice guidelines and independently rated by two experienced physiotherapists using Likert-scale criteria.	ChatGPT demonstrated high clarity (4.85/5) and accuracy (4.62/5) and moderate-to-high guideline alignment, performing best in disease information but showing lower consistency (3.85/5) and weaker agreement in rehabilitation-related recommendations.	Low
Xing Q (2025) [68]	System development and validation study	LLM	Structured (High)	Objective metrics & Experts	Accuracy; Consistency; Interpretability; Effectiveness	A LLM–based framework integrates expert FMS rules with pose-derived keyframe features to perform fine-grained functional movement screen action quality assessment and generate interpretable feedback.	The LLM-based approach achieved high scoring accuracy (0.91) comparable or superior to deep-learning methods while providing fine-grained, interpretable rationales and targeted improvement suggestions for FMS actions.	Low
Sawamura S (2024) [13]	Cross-sectional study	ChatGPT-4	Unstructured (Low to Moderate)	Experts	Accuracy	ChatGPT-4.0 answered 980 Japanese national physical therapist exam questions, with outputs compared against official correct answers.	ChatGPT-4.0 achieved 73.4% overall accuracy and met passing criteria, but performance dropped markedly for practical questions (46.6%) and image/table-based items (35.4%) compared with text-only questions (80.5%).	Low
Erol E (2024) [49]	Cross-sectional study	ChatGPT-3.5	Unstructured (Low)	Physiotherapists	Accuracy; Usability	ChatGPT-3.5 was queried with 23 open-ended questions on core exercise effects, types, and programming, and its responses were evaluated by nine experienced physiotherapists using a 5-point Likert scale.	ChatGPT provided generally satisfactory and accurate information on core exercises (mean score 3.93/5), performing well on general concepts but showing limitations in personalized exercise programming and clinical decision-making.	Low
Biró A (2024) [44]	Qualitative study	Unspecified model	Semi-structured (High)	Objective metrics & Experts	Effectiveness; Personalisation; Safety	Gamification elements combined with Generative AI and reinforcement learning were integrated into team-sport training to generate adaptive scenarios, real-time feedback, and performance-contingent rewards based on athlete data.	The integration of gamification, GenAI, and reinforcement learning increased athlete motivation, engagement, and training adherence, and was associated with improved performance outcomes and team cohesion compared with traditional training approaches.	Moderate

Havers T (2025) [46]	Cross-sectional study	ChatGPT-3.5 (via ChatGPT and via Microsoft Copilot); Gemini (GG)	Semi-structured (High)	Expert coaches	Accuracy; Usability; Consistency; Personalisation; Safety	Three publicly available LLMs were prompted with detailed, standardized inputs to generate 16-week resistance training plans for muscle hypertrophy and maximal strength, which were then evaluated by experienced coaches using 27 evidence-based criteria.	LLM-generated training plans showed overall moderate quality with limited differentiation between hypertrophy and strength goals, with Google Gemini slightly outperforming GPT-3.5 on a few isolated criteria.	Low
Yang J (2025) [54]	Cross-sectional study	ChatGPT-4o; ChatGPT-4.5; ChatGPT-o1; ChatGPT-o3-mini; DeepSeek-R1; DeepSeek-V3	Structured, FITT (High)	Experts	Accuracy; Usability; Consistency; Personalisation; Safety	Six GAI models were prompted with a structured FITT-based instruction to generate 30-day soccer training plans, which were evaluated by expert raters for exercise-prescription quality and by Flesch–Kincaid indices for readability.	All models produced structured soccer-specific programs with moderate and consistent quality (scores 6.0–7.0; ICC=0.79), but showed substantial trade-offs in readability and common deficiencies in evidence citation and individualized health screening.	Low
Masagca R C (2025) [52]	Quasi-experimental study	ChatGPT-3.5	Structured, FITT (High)	Performance tests	Effectiveness; Accuracy; Safety	A 5-week calisthenics training program generated by ChatGPT-3.5 was implemented in untrained collegiate students and compared with a human-made calisthenics program using a quasi-experimental pre–post design.	The AI-generated program significantly improved lower-extremity flexibility and upper-extremity muscular endurance but was inferior to the human-made program for cardiovascular endurance and lower-limb muscular endurance.	Low
Düking P (2024) [50]	Cross-sectional study	ChatGPT 3.0.1	Semi-structured (Low vs. Moderate vs. High)	Expert coaches	Accuracy; Usability; Consistency; Stability; Personalisation; Safety	ChatGPT was prompted with three levels of input information granularity to generate 6-week running training plans for a novice runner, which were evaluated by expert endurance coaches using 22 literature-based quality criteria on a 5-point Likert scale.	ChatGPT-generated running plans were consistently rated as sub-optimal by coaches, but plan quality improved significantly with higher input information granularity.	Low

Washif J A (2024) [10]	Cross-sectional study	ChatGPT-3.5; ChatGPT-4.0	Semi-structured (High)	Experts	Accuracy; Usability; Consistency; Stability; Personalisation; Safety	ChatGPT-3.5 and ChatGPT-4.0 were prompted to generate 12-week periodised resistance-training programmes following structured exercise-prescription inputs, and the outputs were appraised against NSCA guidelines and peer-reviewed literature by domain experts.	ChatGPT produced broadly guideline-consistent, periodised resistance-training programmes, with GPT-4.0 demonstrating greater sensitivity to training status and richer prescription detail than GPT-3.5.	Low
Shin D (2025) [55]	System development and validation study	ChatGPT-4	Semi-structured (High)	Experts, Users & Physiotherapists	Usability; Personalisation; Effectiveness; Accuracy	An LLM-driven conversational agent ("PlanFitting") using GPT-4 with retrieval-augmented generation conducted free-form dialogue to generate weekly exercise plans in IF–THEN "implementation intention" format aligned with ACSM/PA guidelines, evaluated via a user study, intrinsic guideline-compliance checks, and expert FITT-based ratings.	Participants rated PlanFitting as useful and easy to use (TAM), most generated plans met guideline targets (e.g., 83% met ≥150 min/week initially), and experts rated frequency/time above average but flagged weaker "type" composition and some intensity nuances.	Low
Havers T (2025) [47]	Cross-sectional study	ChatGPT-4 (via Microsoft Copilot) and Gemini (1.0 Pro)	Semi-structured (Low vs. High)	Expert coaches	Consistency; Stability; Accuracy; Usability; Personalisation; Safety	Google Gemini and GPT-4 were prompted with two versions of the same hypertrophy RT request by two users to generate 8 resistance-training plans, which were rated by 12 coaching experts on a 5-point Likert scale across literature-derived quality criteria.	The generated plans were highly reproducible across repeated identical prompts (27/28 items showed no differences, p>0.05), while GPT-4 received higher quality ratings than Google Gemini on multiple criteria and higher-information prompts consistently improved perceived plan quality (p=0.000–0.037).	Low
Li J (2025) [56]	System development and validation study	Custom system based on ChatGPT	Semi-structured (High)	Researchers	Accuracy; Usability; Personalisation; Safety	A specialized and fine-tuned ChatGPT model was developed to provide fitness coaching by generating training plans, correcting exercise posture from images, and estimating body composition using limited visual datasets.	ChatGPT could effectively identify incorrect exercise postures and provide general training and nutrition advice, but showed limited personalization, weak visual demonstration quality, and low sensitivity in body fat estimation due to insufficient domain-specific image data.	Low

Vahdati M (2025) [57]	System development and validation study	ChatGPT-4	Structured (High)	Researchers	Personalisation; Safety; Effectiveness	A multi-agent digital twin–based AI fitness coaching system integrated multimodal inputs, OpenPose-based skeletal analysis, GPT-4 reasoning, and short-/long-term memory to deliver personalized workout planning and real-time posture feedback.	The proposed framework enabled adaptive, memory-aware, and multimodal fitness coaching with improved personalization and real-time corrective feedback, demonstrating advantages over single-agent or unimodal AI coaching systems in flexibility, scalability, and user-specific guidance.	Low
Puce L (2026) [48]	Cross-sectional study	ChatGPT-4	Structured, FITT (High)	Expert coaches & Athletes	Accuracy; Usability; Consistency; Stability; Personalisation; Safety	ChatGPT-4 was prompted with a structured, zone-based periodisation template to generate one-week training plans for elite distance swimmers and sprinters during the specific phase, which were evaluated by expert coaches and competitive athletes using Likert-scale ratings across frequency, intensity alignment, and session structure.	Coaches rated AI-generated plans as neutral to moderately acceptable (mean 3.6–3.7/5), with strong performance in low-intensity aerobic zones (A1) but systematic deficiencies in moderate- and high-intensity structuring (A2–B2) and anaerobic frequency, while most plans required human modification.	Low
Fan M (2025) [45]	Quasi-experimental study	Custom system based on GANs and VAEs	Structured (High)	Performance tests & Objective metrics	Effectiveness; Personalisation; Safety	A generative AI system combining GANs and VAEs was used to dynamically generate and adjust personalized HIIT programs based on individual physiological data, wearable-device feedback, and real-time performance monitoring over a 3-month intervention.	AI-designed HIIT significantly improved VO_2max (+15.6%), muscle mass (+8.3%), strength performance (squat +33.3%, bench press +40%), metabolic capacity (+25% METs), and mental health (anxiety −30%, depression −27.3%) compared with baseline.	Moderate
Pajo L S (2025) [51]	Quasi-experimental study	ChatGPT-3.5	Structured, FITT (Low to Moderate)	Performance tests	Effectiveness; Accuracy; Safety	A 10-week volleyball training programme generated by ChatGPT-3.5 using a simple FITT-based prompt was implemented in collegiate volleyball athletes and compared with a human-designed plyometric volleyball programme in a quasi-experimental pre–post design.	The AI-generated programme improved right-side unilateral vertical and horizontal jump performance but failed to enhance bilateral jumping, while the human-made programme produced superior bilateral vertical jump gains.	Low
Wachholz F (2025) [53]	Cross-sectional study	ChatGPT-3.5	Semi-structured (Low to Moderate)	Coaches & Recreational athletes	Usability; Accuracy; Personalisation; Safety; Acceptance	ChatGPT-3.5 was prompted to generate a 12-week beginner half-marathon training plan, which was compared with a human-designed plan using a mixed-methods design combining a TAM+Trust questionnaire in recreational athletes and qualitative quality evaluations by experienced coaches.	About 25% of athletes using training plans relied on AI-generated plans, AI users reported significantly higher trust and acceptance than non-users, and coaches often could not distinguish AI-generated from human-designed plans.	Low

Yang Z (2026) [11]	Cross-sectional study	ChatGPT-4	Structured, FITT (Low vs. High)	Experts	Consistency; Personalisation; Safety; Effectiveness	ChatGPT-4 was repeatedly prompted with simple versus detailed (FITT-structured) inputs to generate one-month training programs for a 15-year-old adolescent, and the outputs were blindly evaluated by 11 sport-science experts for quality and output variability (CV%).	Detailed, structured prompts consistently produced higher expert-rated quality and lower output variability across most dimensions (Personality, Safety, Feasibility, overall score), although repeated identical inputs still yielded residual variability.	Low
Zhang J (2025) [58]	System development and validation study	ChatGPT-3.5-turbo	Structured (High)	Objective metrics & Expert coaches	Accuracy; Consistency; Usability; Effectiveness	A hybrid vision–language framework (ChatMatch) integrating deep-learning-based video encoders with multi-agent LLMs was developed to automatically extract, structure, and interpret racket-sport match videos and answer coaching-relevant analytical queries.	The system achieved high accuracy in meta-feature extraction (location = 0.991, action = 0.902, gesture = 0.950) and demonstrated strong performance in answering general, statistical, and retrieval questions from athletes and coaches.	Low

Supplementary Material
to paper DOI: https://doi.org/10.5114/biolsport.2026.162050

Appendix D Methodological Quality Assessment

(1 = Yes, 0 = No or Unclear)

RCT (n = 2)

JBI Appraisal Item	Philuek P (2025)			Miao C (2025)		
	Response	Score	Comment	Response	Score	Comment
Was true randomization used for assignment of participants to treatment groups?	Yes	1	Simple random sampling method reported for group allocation.	Yes	1	Participants randomly assigned to Group A/B before the first phase (Methods 2.1–2.2).
Was allocation to treatment groups concealed?	Unclear	0	The article does not specify whether allocation concealment (e.g., sealed envelopes) was used.	Unclear	0	Random assignment mentioned, but concealment method (e.g., sealed envelope, third-party) not specified.
Were treatment groups similar at the baseline?	Yes	1	Baseline comparison table shows no significant difference between groups across all measured variables ($p > .05$).	Yes	1	Demographics (age, skill level, experience) were balanced; Table 1 confirmed no significant baseline differences.
Were participants blind to treatment assignment?	No	0	Participants were aware of their group (exercise vs. control); blinding was not feasible.	No	0	Impossible in instructional intervention; students knew whether they were in AI-assisted or traditional sessions.
Were those delivering the treatment blind to treatment assignment?	No	0	Exercise sessions were researcher-supervised; blinding not reported.	No	0	Instructors aware of method used; blinding infeasible in this context.
Were treatment groups treated identically other than the intervention of interest?	Yes	1	Control group continued daily activities; intervention group received structured exercise program as the only difference.	Yes	1	Same videos, environment, duration, and structure; only AI vs. traditional delivery differed.
Were outcome assessors blind to treatment assignment?	Unclear	0	The study does not mention blinding of assessors for post-test measures.	Yes	1	Scoring of tactical comprehension and decision-making conducted by coaches blinded to group allocation.
Were outcomes measured in the same way for treatment groups?	Yes	1	Standardized procedures for BMI, muscle %, and heart rate used pre- and post-intervention in both groups.	Yes	1	Identical tests and instruments (video-based tasks, same scoring rubrics) used for both conditions.
Were outcomes measured in a reliable way	Yes	1	Objective tools used: spirometry, dynamometer, and standard anthropometric assessment.	Yes	1	Three expert raters, standardized scoring rubric, and interrater calibration ensured reliability.
Was follow up complete and if not, were differences between groups in terms of their follow up adequately described and analysed?	Yes	1	All 9 participants completed the 8-week program; no dropout reported.	Yes	1	1 participant withdrew (2.3% attrition) explained; full dataset analyzed.
Were participants analysed in the groups to which they were randomized?	Yes	1	Data were analyzed according to the initial random group assignment.	Yes	1	Data analyzed per assigned order; all valid participants included in analysis.
Was appropriate statistical analysis used?	Yes	1	Non-parametric Wilcoxon tests (appropriate for small samples) were applied for within- and between-group comparisons.	Yes	1	Paired and independent t-tests, normality checks, and effect sizes (Cohen's d) reported.
Was the trial design appropriate and any deviations from the standard RCT design (individual randomization, parallel groups) accounted for in the conduct and analysis of the trial?	Yes	1	The design was clearly described as a pilot RCT; limitations acknowledged regarding small sample and pilot scope.	Yes	1	Crossover design justified; washout phase controlled for order effects; validity verified via pre-test comparisons.
**Totals**	**9**	**9**	**13**	**10**	**10**	**13**

Quasi-experimental (n = 5)

JBI Appraisal Item	Qiu Y (2024)			Kohiyama K (2025)		
	Response	Score	Comment	Response	Score	Comment
Is it clear in the study what is the "cause" and what is the "effect" (i.e. there is no confusion about which variable comes first)?	Yes	1	The intervention (LLM-based autonomous learning vs. teacher-guided learning) is clearly defined as the cause, and motor skill learning outcomes are defined as the effect.	Yes	1	The pretest was conducted before the one-month ChatGPT-based learning period, followed by a posttest, clearly establishing temporal order.
Was there a control group?	Yes	1	A clear control group (CG) taught by PE teachers was used.	No	0	The study employed a single-group pretest–posttest design without a control or comparison group.
Were participants included in any comparisons similar?	Yes	1	Pre-test results showed no significant differences between the two groups (clear, smash, footwork: all p > .05), demonstrating baseline equivalence.	NA	0	No group-level comparisons were performed, as all analyses were based on within-participant pre–post changes.
Were the participants included in any comparisons receiving similar treatment/care, other than the exposure or intervention of interest?	Yes	1	Both groups completed the same 16-week schedule, same skill items, and same testing procedures. The only difference was the mode of instruction (LLM vs. teacher).	Unclear	0	Potential exposure to other learning activities during the study period was not controlled or systematically reported.
Were there multiple measurements of the outcome, both pre and post the intervention/exposure?	Yes	1	The study included pre-tests and post-tests for three badminton skills.	Yes	1	The primary outcome was measured both before and after the intervention using two separate examinations.
Were the outcomes of participants included in any comparisons measured in the same way?	Yes	1	Both groups used the same scales (BSLEAS, BMTEAS) evaluated by expert panels with video recording.	Yes	1	Pre- and post-intervention outcomes were assessed using comparable examination formats and scoring procedures.
Were outcomes measured in a reliable way?	Yes	1	Badminton skill scales were adapted from validated rubrics (RCampus, 2020; Integrating Play Practice Assessment). Expert raters evaluated video recordings.	Unclear	0	Although exam scores were objectively graded, the reliability and equivalence of the test instruments were not formally reported.
Was follow-up complete and if not, were differences between groups in terms of their follow-up adequately described and analyzed?	Yes	1	No attrition or missing data were reported; the full sample (N=80) completed pre- and post-tests.	Yes	1	Follow-up was nearly complete, with only one participant lost and minimal impact on the overall analysis.
Was appropriate statistical analysis used?	Yes	1	Paired t-tests, independent t-tests, and ANCOVA were appropriate for pre–post quasi-experimental design with covariate control.	Yes	1	Paired statistical tests and correlational analyses were appropriately applied to assess pre–post changes and usage patterns.
**Totals**	**9**	**9**	**9**	**5**	**5**	**8**

JBI Appraisal Item	Masagca R C (2025)			Fan M (2025)		
	Response	Score	Comment	Response	Score	Comment
Is it clear in the study what is the “cause” and what is the “effect” (i.e. there is no confusion about which variable comes first)?	Yes	1	Cause = implementation of AI-generated vs human-made calisthenics programs; Effect = changes in flexibility, cardiovascular endurance, and muscular endurance.	Yes	1	Baseline physiological, performance, and mental health measures were collected prior to the three-month AI-designed HIIT intervention, followed by post-intervention assessments.
Was there a control group?	Yes	1	The study included a human-made training group (HMCTP) serving as a control for the AI-generated program (AIGCTP).	No	0	A comparison group following a conventional HIIT program without AI-generated plans was explicitly described.
Were participants included in any comparisons similar?	Yes	1	Both groups (AI vs human program) consisted of untrained college students, matched in sex distribution, height, weight, and BMI.	Unclear	0	Baseline equivalence between the AI-HIIT group and the control group was not clearly reported or statistically demonstrated.
Were the participants included in any comparisons receiving similar treatment/care, other than the exposure or intervention of interest?	Yes	1	Both groups trained three sessions per week for 5 weeks under similar conditions and supervision.	Yes	1	The interventions in each group were relatively standardized.
Were there multiple measurements of the outcome, both pre and post the intervention/exposure?	Yes	1	All dependent variables were measured before and after intervention using validated field tests (Sit and Reach, Step Test, Plank, Wall Sit, Push-up).	Yes	1	Primary outcomes were measured at baseline and again after the three-month intervention period.
Were the outcomes of participants included in any comparisons measured in the same way?	Yes	1	Both groups used identical field-testing protocols and measurement timing (pre/post).	Yes	1	Outcome variables were assessed using the same measurement procedures and instruments before and after the intervention.
Were outcomes measured in a reliable way?	Yes	1	Standardized validated field tests with established reliability were applied for all fitness components.	Yes	1	Objective physiological and performance indicators were reported.
Was follow-up complete and if not, were differences between groups in terms of their follow-up adequately described and analyzed?	Yes	1	All 87 participants completed the intervention; no attrition reported.	Unclear	0	Participant attrition and between-group follow-up completeness were not clearly reported or analyzed.
Was appropriate statistical analysis used?	Yes	1	Appropriate statistical tests: paired t-test (within-group) and independent t-test (between-group), significance level p<.05.	Unclear	0	Descriptive percentage changes were presented, but inferential statistical tests and uncertainty estimates were insufficiently reported.
**Totals**	**9**	**9**	**9**	**5**	**5**	**9**

JBI Appraisal Item	Pajo L S (2025)		
	**Response**	**Score**	**Comment**
Is it clear in the study what is the "cause" and what is the "effect" (i.e. there is no confusion about which variable comes first)?	Yes	1	The intervention (AI-generated vs. human-made volleyball training program) is clearly defined as the cause, and changes in vertical/horizontal jump performance are the effect.
Was there a control group?	Yes	1	Both groups trained for 10 weeks, 3 sessions/week, with identical testing schedules and instructions to avoid extra physical activity.
Were participants included in any comparisons similar?	Yes	1	The human-made training program group serves as a direct control/comparison group.
Were the participants included in any comparisons receiving similar treatment/care, other than the exposure or intervention of interest?	Yes	1	Baseline characteristics (height, weight, BMI, sex distribution) were comparable between groups; no pretest differences were reported.
Were there multiple measurements of the outcome, both pre and post the intervention/exposure?	Yes	1	Pretest and post-test measurements were taken for bilateral and unilateral vertical and horizontal jump performance.
Were the outcomes of participants included in any comparisons measured in the same way?	Yes	1	All instruments used were validated and had high reliability (e.g., vertical jump ICC, broad jump r > .90). Procedures were standardized with multiple trials.
Were outcomes measured in a reliable way?	Yes	1	All 43 participants completed the 10-week training and post-test; no attrition reported.
Was follow-up complete and if not, were differences between groups in terms of their follow-up adequately described and analyzed?	Yes	1	All outcomes were measured using the same standardized tests (vertical jump, single-leg vertical jump, standing broad jump, single-leg broad jump).
Was appropriate statistical analysis used?	Yes	1	Paired t-tests and independent t-tests were appropriate for within- and between-group comparisons. Sample size >30 justified parametric assumptions per authors.
**Totals**	**9**	**9**	**9**

Cross-sectional (n = 31)

JBI Appraisal Item	Chung S M (2024)			Albaloul O (2026)			Saraç H (2025)		
	Response	Score	Comment	Response	Score	Comment	Response	Score	Comment
Were the criteria for inclusion in the sample clearly defined?	Yes	1	The authors explicitly described how 14 frequently asked questions were selected by two diabetes and rehabilitation specialists.	Yes	1	The study explicitly defined the target "sample" as five specified ChatGPT versions and applied a fixed, previously validated basketball CCK test to all versions.	Yes	1	Inclusion of three AI chatbots and expert trainers with defined credentials (≥6 years experience, certified by NASM/EREPS).
Were the study subjects and the setting described in detail?	Yes	1	The study context and evaluators (two physicians from Korea with 7–15 years of experience) were clearly described.	Yes	1	The evaluated ChatGPT versions, testing context, time frame, and assessment setting were described in sufficient methodological detail.	Yes	1	Detailed description of chatbots (version, release year, performance benchmarks) and trainer qualifications; clear design context.
Was the exposure measured in a valid and reliable way?	Yes	1	ChatGPT 4.0 was used consistently for all 14 standardized questions, ensuring reliability of the exposure.	Yes	1	The exposure (ChatGPT version) was clearly operationalised and consistently applied under identical prompting conditions across all models.	Yes	1	Exposure = exercise program generation under standardized prompt; identical input conditions ensured reliability.
Were objective, standard criteria used for measurement of the condition?	Yes	1	The evaluation followed position statements from the American Diabetes Association and the American College of Sports Medicine.	Yes	1	Outcome accuracy was assessed using an established, validated Basketball CCK test with objectively correct multiple-choice answers.	Yes	1	Comparison benchmarks followed ACSM (2021) and NASM (2018, 2021) guidelines — international standards for exercise prescription.
Were confounding factors identified?	Unclear	0	The paper did not explicitly identify possible confounding factors such as subjective bias of raters.	No	0	The study did not explicitly identify potential confounders such as prompt sensitivity, stochastic variation, or model update effects.	Yes	1	Authors recognized possible differences in interpretation, creativity, and model training bias as confounders.
Were strategies to deal with confounding factors stated?	No	0	No strategies for controlling evaluator bias were described, other than reaching consensus through discussion.	No	0	No analytical or design-based strategies (e.g., adjustment, stratification, sensitivity analysis) were reported to address potential confounding.	Unclear	0	No formal control or statistical adjustment (e.g., weighting, inter-rater agreement) was applied to mitigate bias.
Were the outcomes measured in a valid and reliable way?	Yes	1	A 4-point Likert scale was applied for validity, safety, and utility, based on internationally recognized guidelines.	Yes	1	Outcome measurement relied on item-level correctness from a psychometrically validated test and expert verification of explanations.	Yes	1	All outputs (sets, load, heart-rate zones, duration) systematically evaluated by expert panel using established parameters.
Was appropriate statistical analysis used?	Yes	1	Descriptive statistics were used appropriately for this pilot feasibility study.	Yes	1	Cochran's Q test was appropriately applied to compare binary accuracy outcomes across multiple related ChatGPT versions.	No	0	No quantitative analysis beyond descriptive comparison; no inferential statistics performed.
**Totals**	**6**	**6**	**8**	**6**	**6**	**8**	**6**	**6**	**8**

JBI Appraisal Item	Xu Y (2024)			Li G (2025)			Bak M (2024)		
	Response	Score	Comment	Response	Score	Comment	Response	Score	Comment
Were the criteria for inclusion in the sample clearly defined?	Yes	1	The study explicitly stated inclusion and exclusion criteria, including confirmed hypertension with at least one comorbidity, informed consent, and adequate cognitive and language ability.	Yes	1	The study clearly specified inclusion and exclusion criteria, including age range (18–25 years), BMI/body fat thresholds, physical activity level, and PAR-Q clearance.	Yes	1	Inclusion criteria for the 25 validated scenarios (5 health topics × 5 TTM stages) clearly reported and theoretically grounded.
Were the study subjects and the setting described in detail?	Yes	1	Participant characteristics, recruitment period, clinical context, assessment setting, and data collection procedures were described in detail.	Yes	1	The participant's demographic characteristics, anthropometric data, recruitment setting, and contextual background were described in detail.	Yes	1	The evaluated models and experimental design described comprehensively.
Was the exposure measured in a valid and reliable way?	Yes	1	The exposures were clearly operationalised using standardized input data and fixed prompt formats.	Yes	1	The exposure was explicitly defined and generated using standardized inputs and documented prompt protocols.	Yes	1	Exposure = model-generated responses under identical prompts; standardized and repeated with consistency checks across time.
Were objective, standard criteria used for measurement of the condition?	Yes	1	Patient conditions were based on clear clinical diagnoses and objectively measured physiological indicators from validated medical devices.	Yes	1	Baseline condition was determined using objective, standard measures such as BMI, body fat percentage, and PAR-Q screening.	Yes	1	Responses scored using explicit, theory-based rubrics aligned with TTM subprocesses.
Were confounding factors identified?	Unclear	0	The study did not explicitly frame these variables as confounders influencing comparative outcomes.	No	0	The study did not explicitly identify potential confounders such as individual motivation, prior exercise experience, or evaluator bias that could influence expert ratings.	Yes	1	Authors noted potential bias due to model updates, prompt interpretation, and varying health-topic familiarity.
Were strategies to deal with confounding factors stated?	No	0	No statistical adjustment or design-based strategies were reported to control for potential confounding such as disease severity, medication effects, or expert bias.	No	0	No statistical adjustment or design-based strategies were reported to control for confounding, beyond a single-blind expert evaluation.	Yes	1	Reliability ensured by dual coders, intercoder agreement (percent agreement and discussion), and repeated trials.
Were the outcomes measured in a valid and reliable way?	Yes	1	Outcomes were assessed using expert ratings with reported authority coefficients and consistency statistics.	Yes	1	Outcomes were assessed using a structured, theory-based evaluation framework with predefined dimensions and Likert-scale scoring by qualified experts.	Yes	1	Coders independently applied standardized scoring with established reliability metrics; validated rubric ensures construct validity.
Was appropriate statistical analysis used?	Yes	1	Descriptive statistics and inter-expert consistency measures were appropriately applied for expert-based comparative evaluation.	Yes	1	Normality testing and independent-samples t-tests were appropriately applied to compare expert scores between GPT-4 and human coaches.	Yes	1	Descriptive and comparative statistics aligned with study aims; no unnecessary inference applied.

to paper DOI: https://doi.org/10.5114/biolsport.2026.162050

**Totals**	**6**	**6**	**8**	**6**	**6**	**8**	**8**	**8**	**8**

JBI Appraisal Item	Dergaa I (2024)			Vitale J (2025)			Arumilli A (2024)		
	Response	Score	Comment	Response	Score	Comment	Response	Score	Comment
Were the criteria for inclusion in the sample clearly defined?	Yes	1	Inclusion criteria and rationale clearly stated.	Yes	1	The study explicitly defined inclusion criteria for expert participants and clearly specified the evaluated LLMs and predefined FAQs.	Yes	1	Dataset composition explicitly stated with inclusion of emotional and performance dimensions.
Were the study subjects and the setting described in detail?	Yes	1	Detailed profile descriptions simulate typical clinical use scenarios.	Yes	1	The characteristics of the expert panel and the online survey setting were described in detail.	Yes	1	Detailed dataset structure and training/testing procedures described.
Was the exposure measured in a valid and reliable way?	Yes	1	Exposure = GPT-4-generated exercise programs using a standardized, single-prompt procedure; reproducible and transparent.	Yes	1	The exposure was standardized through identical prompts, word limits, timing, and controlled submission procedures.	Yes	1	Exposure = input text prompts processed identically across all models; same tokenization, few-shot setup, and evaluation criteria applied.
Were objective, standard criteria used for measurement of the condition?	Yes	1	Evaluations used the FITT principle, RPE scale, and established ACSM guidelines.	Yes	1	Outcome appropriateness was assessed using predefined Likert-scale criteria and established content validity metrics (CVR, CVI), which are standard methods in expert evaluation studies.	Yes	1	Performance measured via accuracy and cosine-similarity thresholds — clear, replicable metrics.
Were confounding factors identified?	Yes	1	Authors discussed potential confounders such as lack of iterative feedback, evolving model performance, and expert bias.	Yes	1	The authors acknowledged potential sources of variability such as expert subjectivity, disciplinary background differences, and topic complexity (sleep vs. jet lag) as factors influencing ratings.	Yes	1	Authors note differences in model scale, data complexity, and few-shot sensitivity as potential confounders.
Were strategies to deal with confounding factors stated?	Unclear	0	No statistical control or blinding of expert reviewers reported; qualitative synthesis only.	Yes	1	Several strategies were implemented, including rater blinding to LLM identity, randomized presentation order, pilot testing of surveys, repeated assessments (T1/T2), and use of reliability and agreement statistics.	Unclear	0	No statistical controls or normalization across models; performance variability discussed qualitatively only.
Were the outcomes measured in a valid and reliable way?	Yes	1	Expert panel from 20+ countries ensured triangulation and reliability through consensus-based critique.	Yes	1	Outcome measurement reliability was formally assessed using Fleiss' Kappa for inter-rater reliability and Jaccard Similarity Index for intra-rater agreement, with transparent reporting of results.	Yes	1	Standardized accuracy calculations and semantic-similarity evaluation across all models ensure internal reliability.
Was appropriate statistical analysis used?	No	0	Lacked quantitative inter-rater reliability or inferential analysis; relied on narrative evaluation.	Yes	1	The study applied appropriate non-parametric and parametric analyses, aligned with the data structure and distribution.	Yes	1	Comparative descriptive statistics, model-level accuracy tables, and graphical visualizations appropriately used for cross-sectional comparison.

to paper DOI: https://doi.org/10.5114/biolsport.2026.162050

**Totals**	**6**	**6**	**8**	**8**	**8**	**8**	**7**	**7**	**8**

JBI Appraisal Item	Zaleski A L (2024)			Sawamura S (2024)			Akrimi S (2025)		
	Response	Score	Comment	Response	Score	Comment	Response	Score	Comment
Were the criteria for inclusion in the sample clearly defined?	Yes	1	Included all 26 clinical populations with established ACSM guidelines; explicit inclusion rationale.	Yes	1	The study clearly defined the inclusion of evaluation materials as 1,000 questions from the 54th–58th Japanese National Physical Therapist Examinations, with explicit exclusion of officially withdrawn questions.	Yes	1	Three standardized fictional T2DM patient profiles (varying in age, BMI, comorbidities, medication) defined as inclusion cases.
Were the study subjects and the setting described in detail?	Yes	1	Detailed description of AI model (ChatGPT-3.5 Feb 13 version), prompt methodology, and evaluation environment.	Yes	1	The source, structure, language (Japanese), question types (general vs. practical; A-type vs. X2-type), and testing context were described in detail.	Yes	1	Detailed description of fictional patients, prompt versions (1–3), and expert raters (three sport-science professionals with diabetes specialization).
Was the exposure measured in a valid and reliable way?	Yes	1	Exposure = ChatGPT-generated exercise recommendations; standardized prompts and raw performance assessment ensured validity.	Yes	1	The exposure was consistently assessed by inputting all questions under standardized zero-shot conditions and comparing outputs with official answer keys.	Yes	1	Exposure = ChatGPT-4o output generated via three prompt versions; reproducible and version-documented.
Were objective, standard criteria used for measurement of the condition?	Yes	1	Compared against ACSM Guidelines for Exercise Testing and Prescription; used formal coding rubric and Flesch–Kincaid readability.	Yes	1	Accuracy was determined using officially published correct answers and nationally defined exam passing criteria, providing an objective and standardized benchmark.	Yes	1	Assessment followed ADA and ACSM exercise guidelines; predefined quality indicators for safety and training parameters.
Were confounding factors identified?	Yes	1	Authors noted influence of model training data, prompt phrasing, and limited prompt iteration as confounders.	Unclear	0	Potential confounders such as prompt sensitivity, stochastic variability, or model version instability were discussed only briefly and not explicitly framed as confounding factors.	Yes	1	Authors discussed bias due to fictional cases, same raters, and lack of real-patient data.
Were strategies to deal with confounding factors stated?	Unclear	0	Recognized but not statistically controlled; no repeated prompt iterations or sensitivity testing.	No	0	The study did not report analytical or design-based strategies to address potential confounding.	Unclear	0	No blinding or external validation; discussed as limitation only.
Were the outcomes measured in a valid and reliable way?	Yes	1	Dual independent coding with Cohen's κ = 1.0 ensures reliability; validated readability metric used.	Yes	1	Outcome measurement relied on binary correctness against official answers, a highly objective and reproducible outcome definition.	Yes	1	Interrater reliability quantified (ICC = 0.763 → good agreement); ensures internal consistency of expert ratings.
Was appropriate statistical analysis used?	Yes	1	Used descriptive statistics, interrater reliability, and thematic mapping for qualitative insights; suitable for study objectives.	Yes	1	Fisher's exact test and Student's t-test were appropriately applied to compare accuracy across question types, modalities, and lengths.	Yes	1	ICC computation and descriptive frequency analysis (L/M/S scores) appropriate for qualitative-ordinal expert data.

**Totals**	**7**	**7**	**8**	**6**	**6**	**8**	**7**	**7**	**8**

JBI Appraisal Item	Havers T (2025)			Erol E (2024)			Dekerlegand R (2025)		
	Response	Score	Comment	Response	Score	Comment	Response	Score	Comment
Were the criteria for inclusion in the sample clearly defined?	Yes	1	The study explicitly defined the inclusion of evaluation materials as a fixed set of sport- and exercise-related prompts/questions generated under predefined conditions and applied uniformly to the selected LLMs.	Yes	1	Nine physiotherapists (6–11 years experience) selected based on expertise in physiotherapy subfields.	Yes	1	Inclusion criteria (students enrolled in rehabilitation science programs; age ≥18) described in Methods 2.2.
Were the study subjects and the setting described in detail?	Yes	1	The evaluated AI models, application context (sport and exercise science domain), prompt language, evaluation environment, and assessment procedure were described with sufficient methodological detail.	Yes	1	The study described participants' expertise and institutional context (Tokat Gaziosmanpaşa University).	Yes	1	Participants (N=196; from PT, OT, ES, AT, SLP programs at a private university) and setting clearly detailed in Table 1.
Was the exposure measured in a valid and reliable way?	Yes	1	The exposure (LLM-generated responses) was operationalised through standardized prompts and identical input conditions across models, supporting consistency and reproducibility.	Yes	1	Exposure (ChatGPT answers) consistently applied using standardized questions (n=23).	Yes	1	Exposure = "use of GenAI"; measured via TAM/UTAUT-based validated survey instrument reviewed by experts.
Were objective, standard criteria used for measurement of the condition?	Yes	1	Outcome quality was assessed using predefined, theory-informed evaluation criteria and structured scoring rubrics aligned with established domain standards.	Yes	1	Responses rated by 9 physiotherapists on a 5-point Likert scale with standardized scoring.	Yes	1	Standardized Likert-scale questions derived from validated models (TAM, UTAUT).
Were confounding factors identified?	No	0	The study did not explicitly identify potential confounders such as prompt phrasing effects, stochastic output variability, or evaluator background influencing ratings.	Unclear	0	The article did not explicitly address confounding factors (e.g., rater bias, translation effects).	Yes	1	Demographics (age, gender, income, program type) collected; potential confounders acknowledged in Discussion.
Were strategies to deal with confounding factors stated?	No	0	No formal strategies (e.g., repeated sampling, sensitivity analyses, multivariate adjustment, or stratified evaluation) were reported to address potential confounding.	No	0	No mention of controlling inter-rater variability or rater calibration procedures.	No	0	Confounders discussed qualitatively but not statistically controlled (no multivariate modeling).
Were the outcomes measured in a valid and reliable way?	Yes	1	Outcomes were evaluated using a structured rating framework applied consistently across all generated outputs, with expert involvement supporting content validity.	Yes	1	Mean ± SD values reported (3.93±0.46); inter-rater scoring procedure standardized.	Yes	1	Outcomes (perceived usefulness, ease of use, facilitators) assessed via validated scales; pilot tested for reliability.
Was appropriate statistical analysis used?	Yes	1	The statistical methods applied to compare model performance across dimensions were appropriate for the cross-sectional comparative design and outcome scales used.	Yes	1	Descriptive statistics (SPSS 22; mean, SD, range) appropriate for study aims.	Yes	1	Used descriptive statistics and chi-square tests with α=0.05; suitable for categorical survey data.
**Totals**	**6**	**6**	**8**	**6**	**6**	**8**	**7**	**7**	**8**

JBI Appraisal Item	Prosen M (2025)			Wu Q (2025)			Villarreal-Espinosa J B (2024)		
	Response	Score	Comment	Response	Score	Comment	Response	Score	Comment
Were the criteria for inclusion in the sample clearly defined?	Yes	1	Inclusion criteria (undergraduate students from four health-related programs; ≥18 years) explicitly described in Methods 2.1.	Yes	1	Inclusion criteria were clearly described: undergraduate PE majors aged ≥18 with experience using AIGC tools.	Yes	1	Inclusion criteria (top 10 ACL FAQs from AANA and AOSSM institutions) were explicitly described.
Were the study subjects and the setting described in detail?	Yes	1	Setting (University of Primorska, Slovenia) and demographics (gender, field, study year) detailed in Table 1.	Yes	1	Participants were recruited from 10 Chinese universities (comprehensive and sport institutions); demographic details such as gender and grade level were provided (Table 1).	Yes	1	Study setting (orthopedic fellowship institutions) and evaluators (two fellowship-trained surgeons) were well detailed.
Was the exposure measured in a valid and reliable way?	Yes	1	Exposure = "use of generative AI tools"; measured via validated 27-item SAAIHE scale (Cronbach's α=0.90).	Yes	1	Independent variables were based on validated UTAUT and perceived-risk scales, translated and pilot-tested for construct validity.	Yes	1	Exposure (ChatGPT-4 responses to FAQs) was standardized and identical for all questions.
Were objective, standard criteria used for measurement of the condition?	Yes	1	Scale subfactors derived from validated constructs (perceived usefulness, ethics, privacy, etc.).	Yes	1	All constructs were measured with standardized seven-point Likert scales; item loadings exceeded 0.70, confirming convergent validity.	Yes	1	A 5-point Likert scale was used to assess accuracy and justifications were provided.
Were confounding factors identified?	Yes	1	Potential confounders (gender, confidence, frequency/duration of use) identified and tested via regression.	Yes	1	Potential confounders such as gender, education level, and prior AIGC experience were identified and examined for moderating effects.	Unclear	0	Potential bias from raters' subjectivity was acknowledged but not quantitatively controlled.
Were strategies to deal with confounding factors stated?	No	0	Controlled statistically in regression but not in multivariate model adjusting for all covariates simultaneously.	Yes	1	Multi-group analyses were conducted to test gender moderation; results indicated no significant moderation effect.	No	0	No statistical adjustment or control for confounders (e.g., question complexity, rater bias) was applied.
Were the outcomes measured in a valid and reliable way?	Yes	1	Outcomes (attitudes, ethics, perceived usefulness) measured using psychometrically validated scale; factor structure confirmed (EFA/CFA).	Yes	1	Measurement reliability was excellent (Cronbach's $\alpha = 0.903$–0.956; AVE > 0.80; CR > 0.90); the measurement model met all quality thresholds.	Yes	1	Inter-rater agreement was assessed using Cohen's kappa, showing moderate reliability ($\kappa = 0.57$).
Was appropriate statistical analysis used?	Yes	1	Non-parametric and regression analyses appropriate for data type and non-normal distribution.	Yes	1	Partial Least Squares Structural Equation Modeling (PLS-SEM) was used via Smart-PLS 4, including bootstrapping (5000 samples) and tests of R^2 and Q^2 for predictive validity.	Yes	1	Descriptive statistics and weighted kappa were appropriate for cross-sectional accuracy assessment.
**Totals**	**7**	**7**	**8**	**8**	**8**	**8**	**6**	**6**	**8**

JBI Appraisal Item	Gürses Ö A (2025)			Quinn M (2024)			Yang J (2025)		
	Response	Score	Comment	Response	Score	Comment	Response	Score	Comment
Were the criteria for inclusion in the sample clearly defined?	Yes	1	Eligibility criteria for patients (age range, OA grade by Kellgren–Lawrence, diagnostic criteria, exclusions) were explicitly stated.	Yes	1	Inclusion criteria were well defined (15 AAOS CPG prompts; 7 fellowship-trained reviewers).	Yes	1	The study clearly defined the inclusion of six specific GAI models and applied a single, standardized soccer-specific prompt to generate 30-day training plans for evaluation.
Were the study subjects and the setting described in detail?	Yes	1	Participant characteristics, clinical setting, study period, and recruitment flow were clearly reported.	Yes	1	Study context (orthopaedic specialists at multiple institutions) and evaluation structure described clearly.	Yes	1	The evaluated models, prompt structure, assessment context, rater background , and analytical setting were described in sufficient detail.
Was the exposure measured in a valid and reliable way?	Yes	1	The exposures were generated using standardized, structured prompts applied uniformly to all patient profiles.	Yes	1	Exposure (ChatGPT-4 and Gemini responses) standardized using identical prompts and blinding of raters.	Yes	1	Exposure was produced under identical prompt conditions without iterative refinement, allowing consistent cross-model comparison.
Were objective, standard criteria used for measurement of the condition?	Yes	1	Knee OA diagnosis and baseline status were determined using established clinical standards.	Yes	1	Evaluation used a validated 5-point questionnaire across six predefined dimensions.	Yes	1	Training plan quality was assessed using a predefined, theory-informed rubric based on the FITT principle, and readability was measured using established Flesch–Kincaid metrics.
Were confounding factors identified?	Yes	1	The authors acknowledged key potential confounders, including language effects (Turkish prompts), model version updates, response variability, and patient heterogeneity.	Unclear	0	Reviewer variability acknowledged; potential bias discussed but not statistically adjusted.	Unclear	0	Potential confounders such as stochastic output variability, single-run generation, and prompt sensitivity were acknowledged in the limitations but not formally defined as confounding factors.
Were strategies to deal with confounding factors stated?	Unclear	0	Standardization of prompts and consensus physiotherapist programs were used, but no statistical adjustment or sensitivity analysis directly addressed all identified confounders.	No	0	No adjustment (e.g., random effects or multivariate models) to control rater bias.	No	0	No methodological strategies were implemented to control for probabilistic variability across model outputs.
Were the outcomes measured in a valid and reliable way?	Yes	1	Outcome = presence/absence of 50 rehab parameters. Coding was not validated, and inter-rater reliability among coders was not reported. Reliability remains uncertain.	Yes	1	Moderate inter-rater reliability (κ = 0.71) confirms acceptable consistency.	Yes	1	Outcome assessment demonstrated acceptable reliability, with inter-rater reliability quantified using intraclass correlation coefficients (ICC = 0.79).
Was appropriate statistical analysis used?	Yes	1	Chi-square tests were appropriately applied to compare parameter-level agreement frequencies between AI models and physiotherapist consensus.	Yes	1	Descriptive statistics and two-sided t-tests appropriate for comparative analysis.	Yes	1	Descriptive statistics and ICC analyses were appropriate for the cross-sectional, expert-rated comparison of model outputs.
**Totals**	**7**	**7**	**8**	**6**	**6**	**8**	**6**	**6**	**8**

JBI Appraisal Item	Saglam S (2025)			Safran E (2025)			Düking P (2024)		
	**Response**	**Score**	**Comment**	**Response**	**Score**	**Comment**	**Response**	**Score**	**Comment**
Were the criteria for inclusion in the sample clearly defined?	Yes	1	Inclusion criteria were explicit (≥5 years of clinical experience in sports surgery or physiotherapy, basic AI knowledge, active practice).	Yes	1	Inclusion criteria for evaluators (≥8 years of musculoskeletal practice) and predefined CPG-based question set were explicitly described.	Yes	1	The study clearly defined the inclusion of three ChatGPT-generated 6-week running training plans based on explicitly specified levels of input information granularity.
Were the study subjects and the setting described in detail?	Yes	1	Participant demographics, expertise level, and recruitment context were clearly described.	Yes	1	The study clearly defined participants (two physiotherapists) and study context (academic evaluation of ChatGPT GPT-4).	Yes	1	The expert raters' qualifications, coaching experience, number of raters, evaluation context, and the non-clinical, expert-based assessment setting were described in detail.
Was the exposure measured in a valid and reliable way?	Yes	1	Exposure (AI model outputs from GPT-4 and GPT-3.5) was standardized through identical clinical scenarios.	Yes	1	Exposure standardized across 20 controlled prompts submitted in independent sessions.	Yes	1	The exposure was consistently operationalised using predefined prompts and fixed interaction procedures for each input-information condition.
Were objective, standard criteria used for measurement of the condition?	Yes	1	Evaluation used structured 5-point Likert scales for diagnostic accuracy, treatment suitability, surgical technique, and rehabilitation plans.	Yes	1	Assessment criteria were systematically applied using a 5-point Likert scale.	Yes	1	Training plan quality was assessed using a comprehensive, literature-derived framework of 22 predefined criteria reflecting established principles of endurance training prescription.
Were confounding factors identified?	Unclear	0	Potential rater bias (clinicians vs. academics) was discussed but not statistically adjusted beyond subgroup comparison.	Unclear	0	Potential bias acknowledged (same experts who developed and rated questions), but not statistically adjusted.	Yes	1	The authors explicitly discussed potential sources of bias, including input information granularity, rater subjectivity, lack of consensus on "optimal" training plans, and ChatGPT version dependency.
Were strategies to deal with confounding factors stated?	No	0	No multivariate adjustment or control for demographic or professional background differences was performed.	No	0	No corrective design (e.g., independent raters or multivariate analysis) implemented to minimize self-assessment bias.	Unclear	0	Although multiple expert raters, standardized rating scales, and inter-rater reliability analyses were applied, no analytical adjustment or design-based control was implemented to fully address identified confounders.
Were the outcomes measured in a valid and reliable way?	Yes	1	Cronbach's α was calculated (GPT-4 α = 0.478, GPT-3.5 α = 0.234) demonstrating reliability checks of participant ratings.	Yes	1	Weighted kappa statistics were used to test inter-rater reliability (κ = 0.90 for disease info, 0.56 for rehab).	Yes	1	Outcome measurement reliability was formally assessed using Fleiss' Kappa statistics, with transparent reporting of inter-rater agreement for each training plan.
Was appropriate statistical analysis used?	Yes	1	Statistical analyses were appropriate for cross-sectional comparisons.	Yes	1	Descriptive statistics and weighted kappa were appropriate for inter-rater agreement in a cross-sectional design.	Yes	1	Non-parametric Friedman tests with Bonferroni correction were appropriately applied to compare ordinal Likert-scale ratings across training plans.
**Totals**	**6**	**6**	**8**	**6**	**6**	**8**	**7**	**7**	**8**

JBI Appraisal Item	Washif J A (2024)			Havers T (2025)			Yang Z (2026)		
	Response	Score	Comment	Response	Score	Comment	Response	Score	Comment
Were the criteria for inclusion in the sample clearly defined?	Yes	1	The study explicitly defined the evaluated materials as ChatGPT-generated 12-week resistance training programmes.	Yes	1	The study explicitly defined the inclusion of a fixed set of sport- and exercise-related questions/tasks used to evaluate ChatGPT-generated responses under predefined conditions.	Yes	1	The study explicitly defined inclusion criteria for both the prompt provider and the expert panel, with clear eligibility requirements and exclusion conditions.
Were the study subjects and the setting described in detail?	Yes	1	The characteristics of the hypothetical subjects, the ChatGPT versions, and the appraisal context by experienced strength and conditioning experts were clearly described.	Yes	1	The evaluation context, question sources, application domain, and non-clinical, test-based assessment setting were described with sufficient clarity.	Yes	1	The characteristics of the participant, the prompt-generation context, the AI model, and the expert evaluation setting were comprehensively described.
Was the exposure measured in a valid and reliable way?	Yes	1	The exposure was consistently obtained using identical prompts and standardized input procedures across all evaluated items.	Yes	1	The exposure was consistently obtained using identical prompts and standardized input procedures across all evaluated items.	Yes	1	Exposure was operationalised through standardized, repeated prompt inputs conducted in fresh sessions to ensure consistency.
Were objective, standard criteria used for measurement of the condition?	Yes	1	Programme quality was evaluated against established, authoritative standards, providing an objective benchmark for assessment.	Yes	1	Response quality and correctness were assessed against predefined answer keys or expert-derived reference standards grounded in established sport science knowledge.	Yes	1	Training program quality was assessed using a predefined, literature-informed 15-item scale covering Personality, Effectiveness, Safety, and Feasibility.
Were confounding factors identified?	No	0	The study did not explicitly identify potential confounders such as prompt sensitivity, author interpretation bias, or variability inherent in LLM outputs.	Unclear	0	Potential confounders such as prompt wording effects, model stochasticity, and domain difficulty were not explicitly framed as confounding variables.	Yes	1	The authors explicitly acknowledged potential confounders.
Were strategies to deal with confounding factors stated?	No	0	No formal design-based or analytical strategies were reported to control for potential confounding.	No	0	The study did not report methodological or statistical strategies to address potential confounding.	Yes	1	Several strategies were applied, including within-subject crossover design, repeated inputs, session reset to minimise memory effects, and sensitivity analyses.
Were the outcomes measured in a valid and reliable way?	Yes	1	Outcome appraisal was conducted by domain experts with extensive experience.	Yes	1	Outcome assessment relied on structured scoring procedures with clear criteria.	Yes	1	Outcome reliability was supported by structured expert ratings, clear scoring procedures, and consistency checks.
Was appropriate statistical analysis used?	NA	0	The study was primarily descriptive and comparative in nature, without inferential statistical testing, which is appropriate given the exploratory design.	Yes	1	Descriptive statistics and comparative analyses applied were appropriate for the cross-sectional evaluation of model performance.	Yes	1	Repeated-measures ANOVA, CV%, Wilcoxon tests, and Friedman tests were appropriately selected to match the within-subject, cross-sectional comparison of AI

									outputs.
**Totals**	**5**	**5**	**7**	**6**	**6**	**8**	**8**	**8**	**8**

JBI Appraisal Item	Puce L (2026)			Chang S H (2025)			Wachholz F (2025)		
	Response	Score	Comment	Response	Score	Comment	Response	Score	Comment
Were the criteria for inclusion in the sample clearly defined?	Yes	1	Inclusion and exclusion criteria for both coaches and athletes (≥2 years national/international training) were explicitly described.	Yes	1	Inclusion criteria were clearly stated: university and graduate students from a kinesiology college with prior ChatGPT experience.	Yes	1	Inclusion criteria were clearly stated: German proficiency, recreational athletes, residents of Innsbruck; incomplete surveys were excluded.
Were the study subjects and the setting described in detail?	Yes	1	Demographic details and setting (University of Genoa; national competition environment) were well documented.	Yes	1	Demographic information and institutional context (public university in Taiwan) were thoroughly reported.	Yes	1	Age, gender distribution, recruitment procedures, data collection period, and local sports community sampling were all well described.
Was the exposure measured in a valid and reliable way?	Yes	1	Exposure (ChatGPT-4-generated training plans) was standardized using identical prompts for distance vs. sprint specialists.	Yes	1	Exposure variables used previously validated TAM scales; reliability values (α=.76–.92) were reported.	Yes	1	Exposure = use vs. non-use of AI-generated training plans.
Were objective, standard criteria used for measurement of the condition?	Yes	1	Evaluation applied 5-point Likert scales, structured around frequency, intensity alignment, and exercise structure—consistent with validated training criteria.	Yes	1	Survey instruments followed validated constructs; items adapted from peer-reviewed literature and reviewed by experts to ensure validity.	Yes	1	Constructs were adapted from validated TAM scales; reliability (Cronbach's alpha) was reported.
Were confounding factors identified?	Yes	1	The study acknowledged multiple confounders (e.g., coach age, gender, experience, AI familiarity) and modeled them via multivariable regression.	Yes	1	Gender and education level were included as demographic covariates; frequency of use, trust, concerns, and other psychological variables were recognized as potential confounders.	Unclear	0	Potential confounders were not explicitly identified as confounders, although they may influence results.
Were strategies to deal with confounding factors stated?	Yes	1	Regression and ICC analyses quantitatively adjusted for demographic and experiential differences among raters.	Yes	1	Multiple regression models adjusted for demographic variables and key perception constructs to control for confounding.	No	0	No regression or adjustment was used. Only non-parametric group comparisons were conducted without controlling for covariates.
Were the outcomes measured in a valid and reliable way?	Yes	1	Reliability was quantified (ICC=0.609 for coaches, ICC=0.369 for athletes) confirming acceptable internal consistency.	Yes	1	SEDOI and SEDCI were newly developed but reviewed by experts and demonstrated acceptable internal reliability.	Yes	1	TAM constructs had Cronbach's alpha confirming internal consistency; Likert-scale.
Was appropriate statistical analysis used?	Yes	1	ICC, Wilcoxon tests, regression models, and effect sizes (RBC) were appropriately chosen for cross-sectional comparisons.	Yes	1	Descriptive statistics, correlations, and multiple linear regression were appropriate for cross-sectional survey data.	Yes	1	Mann–Whitney U and Wilcoxon tests were appropriate for non-normal ordinal data; effect sizes were reported correctly.
**Totals**	**8**	**8**	**8**	**8**	**8**	**8**	**6**	**6**	**8**

JBI Appraisal Item	Solomon T P J（2025）		
	Response	Score	Comment
Were the criteria for inclusion in the sample clearly defined?	Yes	1	The authors clearly specified that they included publicly available generative AI chatbots from three major providers (OpenAI, Google, Anthropic), and tested basic and advanced models (ChatGPT-4o/4o mini, Gemini 1.5 Flash/Pro, Claude 3.5 Sonnet/Pro). The inclusion of six specific models, and exclusion of Copilot (due to technical constraints), is explicitly described and justified.
Were the study subjects and the setting described in detail?	Yes	1	The "subjects" (chatbots) are described with developer, model name, and level (basic vs advanced), and the setting is detailed: prompts were entered via Google Chrome on MacOS, on two specific dates in October 2024, with OSF protocol registration and clear description of how prompts were handled and outputs stored. Raters (two experienced sports nutritionists) are also described with >15 years of experience, which further contextualizes the setting.
Was the exposure measured in a valid and reliable way?	Yes	1	"Exposure" here corresponds to prompting conditions (simple vs detailed prompts; training vs racing domains; exam questions).
Were objective, standard criteria used for measurement of the condition?	Yes	1	The "condition" (quality of sports nutrition information) was measured against well-established, evidence-based guidelines: ACSM, ISSN, and IAAF consensus statements. The authors pre-defined 20 detailed criteria (Table 3) for training and racing nutrition, and used them to rate accuracy, completeness, clarity, additional info quality, and evidence quality. This constitutes objective, standardised criteria for evaluating chatbot outputs.
Were confounding factors identified?	Yes	1	The authors explicitly discuss several potential confounders and sources of bias, including: Selection bias in criteria and chatbots; AI bias (training data bias, recency bias, lack of transparency); Prompt entry differences between platforms (PDF upload, character limits); Chatbot memory behaviour (differences between OpenAI, Anthropic, Google); Rapid model updates potentially changing performance over time. These are clearly identified in the Limitations section as factors that may influence results.
Were strategies to deal with confounding factors stated?	Yes	1	The authors implemented several strategies: Blinding of raters to chatbot identity (outputs stripped of model-identifying info and coded); Use of an independent prompter to enter prompts and collect outputs; Deleting history, disabling memory, and starting a new chat for each prompt to reduce learning-loop effects; Standardized test–retest timing (two dates within 4 days, no model updates); Pre-registration of protocol and predefined prompting and rating procedures. Although some confounding (e.g., unknown training data) cannot be eliminated, strategies were clearly stated and reasonably robust.
Were the outcomes measured in a valid and reliable way?	Yes	1	Outcome measures include: Accuracy, completeness, clarity, additional info quality, evidence quality (Experiment 1); Multiple-choice exam accuracy (Experiment 2).<br>They used: Explicit Likert scales mapped to guideline-based criteria; Two independent expert raters with good interrater reliability (ICC = 0.893); KR-20 = 0.895 for exam internal consistency; Test–retest reliability analyses for both experiments.<br>These procedures support both validity and reliability of outcome measurements.
Was appropriate statistical analysis used?	Yes	1	The authors conducted a priori power calculations, then used: ICC for interrater reliability; Mixed-model ANOVA / linear mixed models for accuracy (Experiment 1); Logistic mixed models and GLM with binomial distribution for exam accuracy (Experiment 2); Model comparison via log-likelihood, AIC, BIC, plus effect sizes and power. They also treat Likert data appropriately (medians/IQR, and means only when aggregated). Overall, statistical methods are appropriate, well-justified, and clearly reported.
**Totals**	**8**	**8**	**8**

Qualitative study (n = 7)

JBI Appraisal Item	Genç N (2023)			Sapre T (2025)		
	**Response**	**Score**	**Comment**	**Response**	**Score**	**Comment**
Is there congruity between the stated philosophical perspective and the research methodology?	No	0	The study does not explicitly state any underlying philosophical or theoretical perspective to justify the qualitative approach.	Yes	1	The study is explicitly grounded in implementation science and Universal Design for Learning frameworks, which are conceptually aligned with the applied, process-focused qualitative adaptation methodology.
Is there congruity between the research methodology and the research question or objectives?	Yes	1	The exploratory research questions align with a qualitative design aimed at understanding potential applications and concerns regarding ChatGPT.	Yes	1	The objective of systematically adapting an intervention is appropriately addressed through a qualitative rapid-cycle adaptation and expert-driven design process.
Is there congruity between the research methodology and the methods used to collect data?	Yes	1	A semi-structured interview approach is consistent with qualitative inquiry seeking in-depth, descriptive responses.	Yes	1	Data were collected through structured expert meetings, iterative content review, and qualitative synthesis of AI-generated outputs, which are appropriate for an adaptation-focused qualitative study.
Is there congruity between the research methodology and the representation and analysis of data?	Yes	1	The use of content analysis to derive themes is appropriate and consistent with the stated qualitative methodology.	Yes	1	Data are analyzed and represented through transparent thematic categorization, process documentation, and descriptive reporting consistent with qualitative implementation research.
Is there congruity between the research methodology and the interpretation of results?	Yes	1	The findings are interpreted descriptively and thematically rather than causally, which is congruent with qualitative analysis.	Yes	1	Results are interpreted in terms of feasibility, accessibility, and implementation relevance rather than causal inference, aligning with the qualitative design.
Is there a statement locating the researcher culturally or theoretically?	No	0	The researcher does not report their positionality, background, or theoretical stance in relation to the study topic.	Yes	1	The authors explicitly describe the interdisciplinary expert team's professional backgrounds, cultural diversity, and neurodivergent representation, situating the researchers within the research context.
Is the influence of the researcher on the research, and vice- versa, addressed?	No	0	There is no reflection on how the researcher's decisions, assumptions, or interaction with ChatGPT may have shaped the data or interpretations.	Yes	1	The study reflects on how expert judgment, iterative refinement, and human oversight shaped AI outputs and acknowledges reciprocal influence between researchers and the adaptation process.
Are participants, and their voices, adequately represented?	Unclear	0	Although verbatim outputs from ChatGPT are summarized into themes, direct quotations or raw responses are minimally presented.	NA	0	The study does not involve human participants as data sources, focusing instead on program materials, expert input, and AI-generated instructional content.
Is the research ethical according to current criteria or, for recent studies, and is there evidence of ethical approval by an appropriate body?	NA	0	The study states that ethics committee approval was not required, as no human participants were involved.	Yes	1	Ethical considerations are transparently addressed, with justification provided that formal ethics approval was not required due to the absence of human subjects.
Do the conclusions drawn in the research report flow from the analysis, or interpretation, of the data?	Yes	1	The conclusions clearly reflect and synthesize the thematic results generated through content analysis.	Yes	1	Conclusions are directly derived from the documented adaptation process, expert feedback, and qualitative outcomes described in the results.
**Totals**	**5**	**5**	**9**	**9**	**9**	**9**

JBI Appraisal Item	Willms A (2024)			Onan D (2025)		
	**Response**	**Score**	**Comment**	**Response**	**Score**	**Comment**
Is there congruity between the stated philosophical perspective and the research methodology?	Yes	1	The study explicitly adopts an autoethnographic case study approach, which is congruent with its experiential and reflective examination of using ChatGPT in intervention development.	No	0	The study does not articulate any explicit qualitative philosophical stance (e.g., interpretivist, constructivist) underpinning the case-scenario evaluation.
Is there congruity between the research methodology and the research question or objectives?	Yes	1	The objective of exploring feasibility and acceptability is appropriately addressed through an autoethnographic case study design.	Yes	1	The aim of evaluating feasibility and limitations of AI-generated exercise programs is consistent with a qualitative case-scenario analysis.
Is there congruity between the research methodology and the methods used to collect data?	Yes	1	Data collection via researcher field notes, reflective meetings, and documentation of the development process aligns with autoethnographic methodology.	Yes	1	Data collection through structured AI prompting and expert review of generated programs aligns with an exploratory qualitative approach.
Is there congruity between the research methodology and the representation and analysis of data?	Yes	1	Data are thematically organized around acceptability and ease of use, which is consistent with qualitative reflective analysis.	Yes	1	Findings are presented descriptively by case with expert commentary, consistent with qualitative analytical representation.
Is there congruity between the research methodology and the interpretation of results?	Yes	1	Results are interpreted in terms of feasibility, usability, and lessons learned rather than causal effectiveness, matching the qualitative design.	Yes	1	Results are interpreted in terms of clinical plausibility, risks, and professional judgment rather than causal effectiveness.
Is there a statement locating the researcher culturally or theoretically?	Yes	1	The authors explicitly identify themselves as the researchers (AW and SL) and situate their professional roles and experiences within the research process.	No	0	The authors do not explicitly locate themselves theoretically or reflexively beyond listing professional roles.
Is the influence of the researcher on the research, and vice- versa, addressed?	Yes	1	The study clearly reflects on how the researchers' iterative prompting, judgment, and expertise shaped the AI outputs and subsequent interpretations.	Unclear	0	Although expert evaluation shaped interpretation, reflexive discussion of how researchers influenced prompting and analysis is limited.
Are participants, and their voices, adequately represented?	NA	0	No human participants were involved, as the data source consisted of researcher reflections and AI-generated content.	NA	0	No human participants were interviewed, as the data source consisted of AI outputs and expert assessments.
Is the research ethical according to current criteria or, for recent studies, and is there evidence of ethical approval by an appropriate body?	Yes	1	The authors explicitly state that formal ethics approval was not required because no human participants or sensitive data were involved.	Yes	1	The authors clearly state that ethical approval was not required because no human assessment or intervention was conducted.
Do the conclusions drawn in the research report flow from the analysis, or interpretation, of the data?	Yes	1	The conclusions directly synthesize the thematic findings and documented experiences reported in the results section.	Yes	1	Conclusions logically synthesize the case-based expert evaluations reported in the results and discussion sections.
**Totals**	**9**	**9**	**9**	**6**	**6**	**9**

JBI Appraisal Item	McBee J C (2024)			Vieira C (2024)		
	Response	Score	Comment	Response	Score	Comment
Is there congruity between the stated philosophical perspective and the research methodology?	No	0	The study does not explicitly articulate a qualitative philosophical or epistemological stance underpinning the simulation-based approach.	No	0	The study does not explicitly state a qualitative philosophical or epistemological stance guiding the interview-based and design-oriented methodology.
Is there congruity between the research methodology and the research question or objectives?	Yes	1	The objective of evaluating ChatGPT's interdisciplinary competency is appropriately addressed through a simulation-based qualitative case study design.	Yes	1	The objective of exploring AI-assisted narrative personalization based on patient preferences is appropriately addressed through qualitative interviews and design synthesis.
Is there congruity between the research methodology and the methods used to collect data?	Yes	1	Data collection via structured prompt-driven simulations and recorded panel discussions aligns with the exploratory qualitative methodology.	Yes	1	Semi-structured patient interviews are suitable for eliciting preferences, experiences, and narrative interests relevant to qualitative design research.
Is there congruity between the research methodology and the representation and analysis of data?	Yes	1	Findings are organized thematically by topic domain and expert role, consistent with qualitative descriptive analysis.	Yes	1	Interview data are thematically summarized and translated into patient profiles and narrative design elements, consistent with qualitative descriptive analysis.
Is there congruity between the research methodology and the interpretation of results?	Yes	1	Results are interpreted in terms of conceptual strengths, limitations, and educational implications rather than causal effectiveness.	Yes	1	Results are interpreted in terms of engagement, immersion, and design relevance rather than clinical causality, aligning with the exploratory qualitative approach.
Is there a statement locating the researcher culturally or theoretically?	No	0	The authors do not explicitly locate themselves theoretically or reflexively beyond institutional and disciplinary affiliations.	No	0	The authors do not provide reflexive statements regarding their positionality, assumptions, or theoretical orientation in relation to participants or analysis.
Is the influence of the researcher on the research, and vice- versa, addressed?	Unclear	0	Although the role of the human operator in steering simulations is described, reflexive discussion of researcher influence on interpretation is limited.	No	0	There is no explicit reflection on how researcher decisions, interview framing, or AI prompting may have influenced the findings or narrative outcomes.
Are participants, and their voices, adequately represented?	NA	0	No human participants were involved, as the data source consisted of AI-generated panel responses.	Yes	1	Participants' preferences and perspectives are clearly reflected through summarized interview findings and illustrative AI-generated narrative excerpts.
Is the research ethical according to current criteria or, for recent studies, and is there evidence of ethical approval by an appropriate body?	Yes	1	The study clearly states that institutional review was not required because it did not constitute human-subjects research.	Unclear	0	Although anonymization procedures are described, explicit reporting of ethical approval or exemption by an ethics committee is absent.
Do the conclusions drawn in the research report flow from the analysis, or interpretation, of the data?	Yes	1	Conclusions are directly derived from the thematic synthesis of simulation results and documented observations.	Yes	1	The conclusions logically derive from the reported interview findings and the AI-assisted narrative design process.
**Totals**	**6**	**6**	**9**	**6**	**6**	**10**

JBI Appraisal Item	Biró A (2024)		
	Response	Score	Comment
Is there congruity between the stated philosophical perspective and the research methodology?	No	0	The paper does not state an explicit qualitative philosophical or epistemological perspective underpinning the mixed and exploratory approach.
Is there congruity between the research methodology and the research question or objectives?	Yes	1	The objective of examining motivation and engagement through gamification and GenAI is consistent with the use of qualitative assessments alongside performance data.
Is there congruity between the research methodology and the methods used to collect data?	Yes	1	Qualitative feedback from athletes and coaches is appropriately used to assess engagement and motivation within the proposed framework.
Is there congruity between the research methodology and the representation and analysis of data?	Unclear	0	Although qualitative comments are reported, the analytic procedures for these data are not clearly described or systematized.
Is there congruity between the research methodology and the interpretation of results?	Yes	1	Qualitative findings are interpreted descriptively in relation to motivation, engagement, and team dynamics rather than causal inference.
Is there a statement locating the researcher culturally or theoretically?	No	0	The author does not provide reflexive discussion of personal, cultural, or theoretical positioning in relation to the study.
Is the influence of the researcher on the research, and vice- versa, addressed?	No	0	There is no explicit reflection on how researcher decisions, model design, or interpretation may have influenced the qualitative findings.
Are participants, and their voices, adequately represented?	Yes	1	Perspectives are summarized.
Is the research ethical according to current criteria or, for recent studies, and is there evidence of ethical approval by an appropriate body?	Yes	1	Depersonalized datasets are mentioned.
Do the conclusions drawn in the research report flow from the analysis, or interpretation, of the data?	Yes	1	The conclusions align with the reported quantitative trends and summarized qualitative observations on motivation and engagement.
**Totals**	**6**	**6**	**10**

Summary

Study Type	Study #	Study ID	Total YES	Evaluated Items	Percent YES	Risk of bias	Notes
RCT	1	Philuek P (2025)	9	13	69%	Moderate	
RCT	2	Miao C (2025)	10	13	77%	Low	
Quasi-experimental	1	Qiu Y (2024)	9	9	100%	Low	
Quasi-experimental	2	Kohiyama K (2025)	5	8	63%	Moderate	
Quasi-experimental	3	Masagca R C (2025)	9	9	100%	Low	
Quasi-experimental	4	Fan M (2025)	5	9	56%	Moderate	
Quasi-experimental	5	Pajo L S (2025)	9	9	100%	Low	
Cross-sectional	1	Chung S M (2024)	6	8	75%	Low	
Cross-sectional	2	Albaloul O (2026)	6	8	75%	Low	
Cross-sectional	3	Saraç H (2025)	6	8	75%	Low	
Cross-sectional	4	Xu Y (2024)	6	8	75%	Low	
Cross-sectional	5	Li G (2025)	6	8	75%	Low	
Cross-sectional	6	Bak M (2024)	8	8	100%	Low	
Cross-sectional	7	Dergaa I (2024)	6	8	75%	Low	
Cross-sectional	8	Vitale J (2025)	8	8	100%	Low	
Cross-sectional	9	Arumilli A (2024)	7	8	88%	Low	
Cross-sectional	10	Zaleski A L (2024)	7	8	88%	Low	
Cross-sectional	11	Sawamura S (2024)	6	8	75%	Low	
Cross-sectional	12	Akrimi S (2025)	7	8	88%	Low	
Cross-sectional	13	Havers T (2025)	6	8	75%	Low	
Cross-sectional	14	Erol E (2024)	6	8	75%	Low	
Cross-sectional	15	Dekerlegand R (2025)	7	8	88%	Low	
Cross-sectional	16	Prosen M (2025)	7	8	88%	Low	
Cross-sectional	17	Wu Q (2025)	8	8	100%	Low	
Cross-sectional	18	Villarreal-Espinosa J B (2024)	6	8	75%	Low	
Cross-sectional	19	Gürses Ö A (2025)	7	8	88%	Low	
Cross-sectional	20	Quinn M (2024)	6	8	75%	Low	
Cross-sectional	21	Yang J (2025)	6	8	75%	Low	
Cross-sectional	22	Saglam S (2025)	6	8	75%	Low	
Cross-sectional	23	Safran E (2025)	6	8	75%	Low	

Cross-sectional	24	Düking P (2024)	7	8	88%	Low
Cross-sectional	25	Washif J A (2024)	5	7	71%	Low
Cross-sectional	26	Havers T (2025)	6	8	75%	Low
Cross-sectional	27	Yang Z (2026)	8	8	100%	Low
Cross-sectional	28	Puce L (2026)	8	8	100%	Low
Cross-sectional	29	Chang S H (2025)	8	8	100%	Low
Cross-sectional	30	Wachholz F (2025)	6	8	75%	Low
Cross-sectional	31	Solomon T P J（2025）	8	8	100%	Low
Qualitative	1	Genç N (2023)	5	9	56%	Moderate
Qualitative	2	Sapre T (2025)	9	9	100%	Low
Qualitative	3	Willms A (2024)	9	9	100%	Low
Qualitative	4	Onan D (2025)	6	9	67%	Moderate
Qualitative	5	McBee J C (2024)	6	9	67%	Moderate
Qualitative	6	Vieira C (2024)	6	10	60%	Moderate
Qualitative	7	Biró A (2024)	6	10	60%	Moderate

Appendix E LDA Modeling Code

Code to generate Perplexity_Coherence_raw.csv 、 Perplexity_Coherence_combined.csv 、 Perplexity_Coherence_dual_combined_bestK.png 、 Model_Score_vs_K.png 、 LDA_bestK*_topics.xlsx 、 Topic_Keyword_Network.png:

```
# -*- coding: utf-8 -*-
"""

LDA 自动选 K（困惑度 + 一致性）、主题关键词导出、三张图 + 网络图

"""

import os, re
import numpy as np
import pandas as pd
from tqdm import tqdm
import matplotlib.pyplot as plt
import networkx as nx
from matplotlib import cm
from sklearn.feature_extraction.text import CountVectorizer, ENGLISH_STOP_WORDS
from sklearn.decomposition import LatentDirichletAllocation
from gensim.corpora import Dictionary
from gensim.models.coherencemodel import CoherenceModel

# =============== 参数 ===============
CSV_PATH = "AI_in_PE_texts.csv"
TOP_N_WORDS = 15
W_COH = 0.5
W_PERP = 0.5
K_MIN, K_MAX = 2, 8

CUSTOM_STOPWORDS = {
    "chatgpt", "gpt", "llm", "ai", "artificial", "intelligence",
    "sport", "sports", "exercise", "exercises", "training",
    "physical", "students", "student", "participant", "participants",
    "program", "programs", "data", "clinical", "study"
}
# ====================================


def plot_topic_keyword_network(lda_model, vocab, n_top_words, out_path):
    """绘制主题-关键词网络图"""

    topics_words, topics_weights = [], []
    for comp in lda_model.components_:
        idx = comp.argsort()[:-n_top_words - 1:-1]
        topics_words.append([vocab[i] for i in idx])
        topics_weights.append([float(comp[i]) for i in idx])
```

```
    G = nx.Graph()
    for ti in range(len(topics_words)):
        G.add_node(f"Topic-{ti+1}", kind="topic")

    for ti, (words, weights) in enumerate(zip(topics_words, topics_weights), start=1):
        tname = f"Topic-{ti}"
        for w, wt in zip(words, weights):
            if w not in G:
                G.add_node(w, kind="word", weight=wt)
            G.add_edge(tname, w, weight=wt)

    pos = nx.spring_layout(G, k=0.7, seed=42)

    topic_nodes = [n for n, d in G.nodes(data=True) if d.get("kind") == "topic"]
    word_nodes = [n for n, d in G.nodes(data=True) if d.get("kind") == "word"]

    wts = [G.nodes[n].get("weight", 1.0) for n in word_nodes]
    w_min, w_max = min(wts), max(wts)
    sizes = [300 * (0.3 + 0.7*(G.nodes[n].get("weight",1)-w_min)/(w_max-w_min+1e-12)) for n in word_nodes]

    cmap = cm.get_cmap("tab10", len(topic_nodes))
    colors = [cmap(i) for i in range(len(topic_nodes))]

    plt.figure(figsize=(12, 8))
    nx.draw_networkx_nodes(G, pos, nodelist=topic_nodes, node_shape="s",
                           node_color=colors, node_size=1200, alpha=0.95)
    nx.draw_networkx_nodes(G, pos, nodelist=word_nodes, node_shape="o",
                           node_color="#f08080", node_size=sizes, alpha=0.85)
    nx.draw_networkx_edges(G, pos, alpha=0.4)
    nx.draw_networkx_labels(G, pos, font_size=9)

    plt.title("Topic–Keyword Network", fontsize=16)
    plt.axis("off")
    plt.tight_layout()
    os.makedirs("lda_outputs", exist_ok=True)
    plt.savefig(out_path, dpi=300)
    plt.close()


def main():
    os.makedirs("lda_outputs", exist_ok=True)

    # ---------- 1. 读入 & 清洗 ----------
    df = pd.read_csv(CSV_PATH, encoding="utf-8-sig")
    df["text"] = df["Title"].fillna("") + " " + df["Abstract"].fillna("") + " " + df["Keywords"].fillna("")

    def clean(s):
```

```
    s = re.sub(r"[^A-Za-z]+", " ", str(s)).lower()
    toks = [w for w in s.split() if len(w) > 2]
    return " ".join(toks)

texts = [clean(t) for t in tqdm(df["text"].tolist(), desc="Cleaning")]


# ---------- 2. 停用词 ----------

stop_words = list(ENGLISH_STOP_WORDS.union(CUSTOM_STOPWORDS))

# ---------- 3. 向量化 ----------
cv = CountVectorizer(
    stop_words=stop_words,
    max_df=0.4,
    min_df=2,
    ngram_range=(1, 2),
    max_features=6000
)
X = cv.fit_transform(texts)
vocab = cv.get_feature_names_out()

analyzer = cv.build_analyzer()
tokens_list = [analyzer(t) for t in texts]
dictionary = Dictionary(tokens_list)


# ---------- 4. 评估不同 K ----------

def evaluate_k(k):
    lda = LatentDirichletAllocation(
        n_components=k,
        random_state=42,
        learning_method="batch",
        max_iter=100
    )
    lda.fit(X)

    score = lda.score(X)
    perp = lda.perplexity(X)

    topics = []
    for comp in lda.components_:
        idx = comp.argsort()[:-TOP_N_WORDS - 1:-1]
        topics.append([vocab[i] for i in idx])

    coh = CoherenceModel(
        topics=topics, texts=tokens_list,
        dictionary=dictionary, coherence="c_v"
    ).get_coherence()
```

```
    return perp, coh, score, lda, topics

records = []
models = {}
topic_dict = {}

for k in tqdm(range(K_MIN, K_MAX+1), desc="Grid K"):
    perp, coh, score, lda_model, topics = evaluate_k(k)
    records.append((k, score, perp, coh))
    models[k] = lda_model
    topic_dict[k] = topics
    print(f"K={k} | Score={score:.2f} | Perplexity={perp:.2f} | Coherence={coh:.4f}")

# ---------- 5. 综合指标 ----------

df_k = pd.DataFrame(records, columns=["K", "Score", "Perplexity", "Coherence"])
df_k.to_csv("lda_outputs/Perplexity_Coherence_raw.csv", index=False)

coh_norm = (df_k["Coherence"] - df_k["Coherence"].min()) / (df_k["Coherence"].max() - df_k["Coherence"].min() + 1e-12)
inv_perp = df_k["Perplexity"].max() - df_k["Perplexity"]
perp_norm = (inv_perp - inv_perp.min()) / (inv_perp.max() - inv_perp.min() + 1e-12)

df_k["coh_norm"] = coh_norm
df_k["perp_norm"] = perp_norm
df_k["combined"] = W_COH * coh_norm + W_PERP * perp_norm
df_k.to_csv("lda_outputs/Perplexity_Coherence_combined.csv", index=False)

bestK = int(df_k.loc[df_k["combined"].idxmax(), "K"])

print("\n☆ 最优 K =", bestK)


# ---------- 6. 图1：Perplexity & Coherence ----------

fig, ax1 = plt.subplots(figsize=(7, 5))
ax1.set_xlabel("Number of Topics (K)")
ax1.set_ylabel("Perplexity (lower=better)", color="tab:blue")
ax1.plot(df_k["K"], df_k["Perplexity"], marker="o", color="tab:blue", linewidth=2)
ax1.tick_params(axis="y", labelcolor="tab:blue")
ax1.grid(True, linestyle="--", alpha=0.5)

ax2 = ax1.twinx()
ax2.set_ylabel("Coherence (higher=better)", color="tab:pink")
ax2.plot(df_k["K"], df_k["Coherence"], marker="s", color="tab:pink", linewidth=2)
ax2.tick_params(axis="y", labelcolor="tab:pink")

best_row = df_k[df_k["K"] == bestK].iloc[0]
ax1.scatter([bestK], [best_row["Perplexity"]], color="tab:blue", s=80, zorder=5)
ax2.scatter([bestK], [best_row["Coherence"]], color="tab:pink", s=80, zorder=6)
ax2.text(
```

```
    bestK,
    best_row["Coherence"] - 0.02,
    s=f"Best K={bestK}\nCoh={best_row['Coherence']:.3f}  Perp={best_row['Perplexity']:.1f}",
    color='black',
    fontsize=9,
    weight='bold',
    va='top',
    ha='center'
)

plt.title("Perplexity & Coherence vs K (Best K Highlighted)")
fig.tight_layout()
plt.savefig("lda_outputs/Perplexity_Coherence_dual_combined_bestK.png", dpi=300)
plt.close()

# ---------- 7. 图 2 ：Score vs K ----------

plt.figure(figsize=(7, 5))
plt.plot(df_k["K"], df_k["Score"], marker="o", linewidth=2)
plt.xlabel("Number of Topics (K)")
plt.ylabel("Model Score (log-likelihood)")
plt.title("LDA Model Score vs K")
plt.grid(True, linestyle="--", alpha=0.5)
plt.tight_layout()
plt.savefig("lda_outputs/Model_Score_vs_K.png", dpi=300)
plt.close()

# ---------- 8. 导出最优 K 主题 ----------

best_topics = topic_dict[bestK]
df_topics = pd.DataFrame({
    "Topic": [f"Topic {i}" for i in range(len(best_topics))],
    "Keywords": [", ".join(words) for words in best_topics]
})
df_topics.to_excel(f"lda_outputs/LDA_bestK{bestK}_topics.xlsx", index=False)

# ---------- 9. 图 3 ：主题-关键词网络图 ----------

plot_topic_keyword_network(
    lda_model=models[bestK],
    vocab=vocab,
    n_top_words=TOP_N_WORDS,
    out_path="lda_outputs/Topic_Keyword_Network.png"
)

print("\n✅ 已生成以下文件（在 lda_outputs 文件夹中）：")

print(" - Perplexity_Coherence_raw.csv")
print(" - Perplexity_Coherence_combined.csv")
```

```
    print(" - Perplexity_Coherence_dual_combined_bestK.png")
    print(" - Model_Score_vs_K.png")
    print(" - LDA_bestK*_topics.xlsx")
    print(" - Topic_Keyword_Network.png")


if __name__ == "__main__":
    main()
```
